# Exposure of pelagic seabirds to *Toxoplasma gondii* in the Western Indian Ocean points to an open sea dispersal of this terrestrial parasite

**DOI:** 10.1371/journal.pone.0255664

**Published:** 2021-08-18

**Authors:** Marie-Lazarine Poulle, Matthieu Le Corre, Matthieu Bastien, Elsa Gedda, Chris Feare, Audrey Jaeger, Christine Larose, Nirmal Shah, Nina Voogt, Byron Göpper, Erwan Lagadec, Gérard Rocamora, Régine Geers, Dominique Aubert, Isabelle Villena, Camille Lebarbenchon

**Affiliations:** 1 Epidémio-Surveillance et Circulation des Parasites dans les Environnements (ESCAPE), EA 7510, CAP SANTE, Université de Reims Champagne Ardenne, Reims, France; 2 CERFE, Université de Reims Champagne-Ardenne, Boult-aux-Bois, France; 3 UMR Ecologie marine tropicale des océans Pacifique et Indien (ENTROPIE), CNRS IRD, IFREMER, Université de Nouvelle-Calédonie, Université de la Réunion, Saint Denis, La Réunion, France; 4 Université de La Réunion, UMR Processus Infectieux en Milieu Insulaire Tropical (PIMIT), INSERM 1187, CNRS 9192, IRD 249, Saint Denis, La Réunion, France; 5 WildWings Bird Management, Haslemere, Surrey, United Kingdom; 6 Center for Environment and Education, Nature Seychelles, Roche Caïman, Mahé, Seychelles; 7 Cousine Island, Seychelles; 8 Island Biodiversity and Conservation Centre, University of Seychelles, Anse Royale, Seychelles; 9 Island Conservation Society, Mahé, Seychelles; 10 Laboratoire de Parasitologie-Mycologie, Centre National de Référence de la Toxoplasmose, Centre de Ressources Biologiques *Toxoplasma*, CHU Reims, Reims, France; MARE – Marine and Environmental Sciences Centre, PORTUGAL

## Abstract

*Toxoplasma gondii* is a protozoan parasite that uses felids as definitive hosts and warm-blooded animals as intermediate hosts. While the dispersal of *T*. *gondii* infectious oocysts from land to coastal waters has been well documented, transmission routes to pelagic species remain puzzling. We used the modified agglutination test (MAT titre ≥ 10) to detect antibodies against *T*. *gondii* in sera collected from 1014 pelagic seabirds belonging to 10 species. Sampling was carried out on eight islands of the Western Indian Ocean: Reunion and Juan de Nova (colonized by cats), Cousin, Cousine, Aride, Bird, Europa and Tromelin islands (cat-free). Antibodies against *T*. *gondii* were found in all islands and all species but the great frigatebird. The overall seroprevalence was 16.8% [95% CI: 14.5%-19.1%] but significantly varied according to species, islands and age-classes. The low antibody levels (MAT titres = 10 or 25) detected in one shearwater and three red-footed booby chicks most likely resulted from maternal antibody transfer. In adults, exposure to soils contaminated by locally deposited oocysts may explain the detection of antibodies in both wedge-tailed shearwaters on Reunion Island and sooty terns on Juan de Nova. However, 144 adults breeding on cat-free islands also tested positive. In the Seychelles, there was a significant decrease in *T*. *gondii* prevalence associated with greater distances to cat populations for species that sometimes rest on the shore, i.e. terns and noddies. This suggests that oocysts carried by marine currents could be deposited on shore tens of kilometres from their initial deposition point and that the number of deposited oocysts decreases with distance from the nearest cat population. The consumption of fishes from the families Mullidae, Carangidae, Clupeidae and Engraulidae, previously described as *T*. *gondii* oocyst-carriers (i.e. paratenic hosts), could also explain the exposure of terns, noddies, boobies and tropicbirds to *T*. *gondii*. Our detection of antibodies against *T*. *gondii* in seabirds that fish in the high sea, have no contact with locally contaminated soils but frequent the shores and/or consume paratenic hosts supports the hypothesis of an open-sea dispersal of *T*. *gondii* oocysts by oceanic currents and/or fish.

## Introduction

The land-to-sea transport of the free infective forms of zoonotic protozoa (oocysts or cyst), dispersed with the faeces of humans, pets and farm animals has a growing negative impact on public health and marine life [1, 2]. While several studies have been carried out on faecal contamination of the coastal environment with *Cryptosporidium*, *Giardia* and *Toxoplasma* [3–5], less attention has been paid to the open ocean, resulting in a critical lack of information on the transmission routes of protozoan parasites to pelagic species. This gap is particularly problematic for *Toxoplasma gondii* because this apicomplexan parasite is currently emerging as an important pathogen in aquatic systems [6–8]. *Toxoplasma gondii* is responsible for toxoplasmosis, one of the most common parasitic infections of warm-blooded animals, including humans [9]. The finding of acute toxoplasmosis and the detection of antibodies against *T*. *gondii* in marine mammals in the Eastern, Central and Western Pacific [10], the Canadian Arctic [11], the Northeastern and Western Atlantic [10, 12], the Philippine archipelago [13] and the Mediterranean Sea [14] suggests a worldwide contamination of marine habitats.

The environmental contamination with *T*. *gondii* necessarily comes from felids since domestic cat, *Felis catus*, and wild felids are the only known definitive hosts in which the sexual multiplication of *T*. *gondii* occurs, resulting in the faecal shedding of oocysts into the environment [15]. These oocysts are highly resistant and can remain infective in soils for months [16–18]. All warm-blooded animals can be intermediate host for *T*. *gondii* [9]. Once the oocysts have been ingested by a mammal or a bird, the development of *T*. *gondii* continues until the formation of infecting tissue cysts [19]. These cysts can persist lifelong in the host and IgG antibodies probably do the same [9, 20]. The prevalence of antibodies to *T*. *gondii* is therefore generally higher in adult than in juvenile populations, both in wild birds [21] and in wild and domestic mammals [22, 23] due to a longer period of exposure which increases the likelihood of infection.

Acute toxoplasmosis is rarely reported in terrestrial birds and mammals that have co-evolved with felids and their parasites, but wildlife species recently exposed to *T*. *gondii* can be severely affected [24, 25]. Fatal toxoplasmosis is notably reported in marsupials and native terrestrial birds in Australia [26, 27] and Hawaii [28] where *T*. *gondii* was absent until the introduction of the domestic cat. Meningoencephalitis associated with *T*. *gondii* also results in morbidity and mortality in free-ranging sea otters, *Enhydra lutris* [29], sea lions, *Zalophus californianus* [30] and dolphins [14], especially when associated with poly-parasitism or environmental pollutants [31, 32]. As a result, *T*. *gondii* is considered a pathogen of concern for several marine mammal species [33].

Recent molecular epidemiology studies provide evidence that freshwater can carry *T*. *gondii* oocysts from terrestrial to marine coastal habitats [34–36]. The dilution of oocysts to a low concentration in the marine environment is compensated by their ability to survive and to remain infectious for several months in seawater [37], by their filtration and bio-accumulation in marine bivalves [38, 39] and their capture by planktonic animals that are a major source of food for fish and invertebrates [7, 40]. Oocysts can also adhere to kelp grazed by marine snails, resulting in a high concentration of oocysts in their faecal pellets [41, 42]. In addition, infectious oocysts can be transported in the digestive tract of migratory filter feeding fish [43]. The consumption of marine fishes and invertebrates that carry *T*. *gondii* oocysts (i.e. paratenic hosts) may therefore be considered as responsible for the contamination of coastal marine predators like sea otters [34, 44, 45], coastal dolphins foraging in Atlantic Ocean bays and Mediterranean coasts [46, 47] or beluga whales and seals from the St. Lawrence stream, Canada [48, 49]. Antibodies against *T*. *gondii* have also been detected far away from potential contamination sources by cats as in Weddell seals, *Leptonychotes weddellii*, and elephant seals, *Mirounga leonina*, sampled in the Antarctic Peninsula [50, 51], or in pelagic dolphins [14, 46], as well as in pelagic seabirds breeding on felid-free islands [52–54]. In all these cases, the transport of infectious oocysts by marine currents or by fish have been mentioned as the two likely routes of transmission of *T*. *gondii* to pelagic species but without evidence of the involvement of one and/or the other in the exposure of the species studied.

The present study aims at exploring the variability in exposure to *T*. *gondii* in ten pelagic seabird species breeding in the Western Indian Ocean in order to elucidate the routes of transmission of this protozoan to “offshore” species. Pelagic seabirds are good models for assessing the relative importance of *T*. *gondii* transmission routes in pelagic environments since they spend most of their time far at sea, rarely venturing close to land except to breed, and obtain their food and most of their drinking water from fish, squids and other marine invertebrates [55]. Serum samples were obtained from seabirds breeding on eight islands, two of which are colonized by cats and six are felid-free. Based on this sampling, we tested whether the prevalence of *T*. *gondii* in seabirds varied according to age-class, species, islands and nesting habits. In particular, we expected a lower prevalence on cat-free islands than on islands where birds are exposed to oocysts dispersed by resident cats, and a higher prevalence in ground nesters than in tree-nesters as the latter are assumed to be less exposed to oocyst-contaminated soil. For species that frequent the coastline (i.e. terns and noddies), we expected a higher prevalence on islands close to cat populations than on remote islands, as the latter are assumed to receive lower numbers of oocysts on their shores. Finally, based on the literature on seabird diet, we discussed the relationship between the prevalence of *T*. *gondii* in seabirds and their consumption of paratenic-host fish.

## Materials and methods

### Ethical approval

Procedures were evaluated and approved by an ethic committee (agreement # A974 001, Comité d’éthique du CYROI # 114; Cyclotron Réunion Océan Indien, Sainte Clotilde, La Réunion, France), and authorized by The French Ministry of Education and Research (reference number APAFIS#3719-2016012110233597v2). Sample collection on Reunion Island, Europa, Juan de Nova and Tromelin was conducted under the approval of the Direction de l’Environnement, de l’Aménagement et du Logement de la Réunion and the Terres Australes and Antarctiques Françaises. Fieldwork and collection of biological material in the Seychelles were approved by the Seychelles Bureau of Standards and the Seychelles Ministry of Environment, Energy and Climate Change.

### Study sites and sample collection

**Fig 1:**
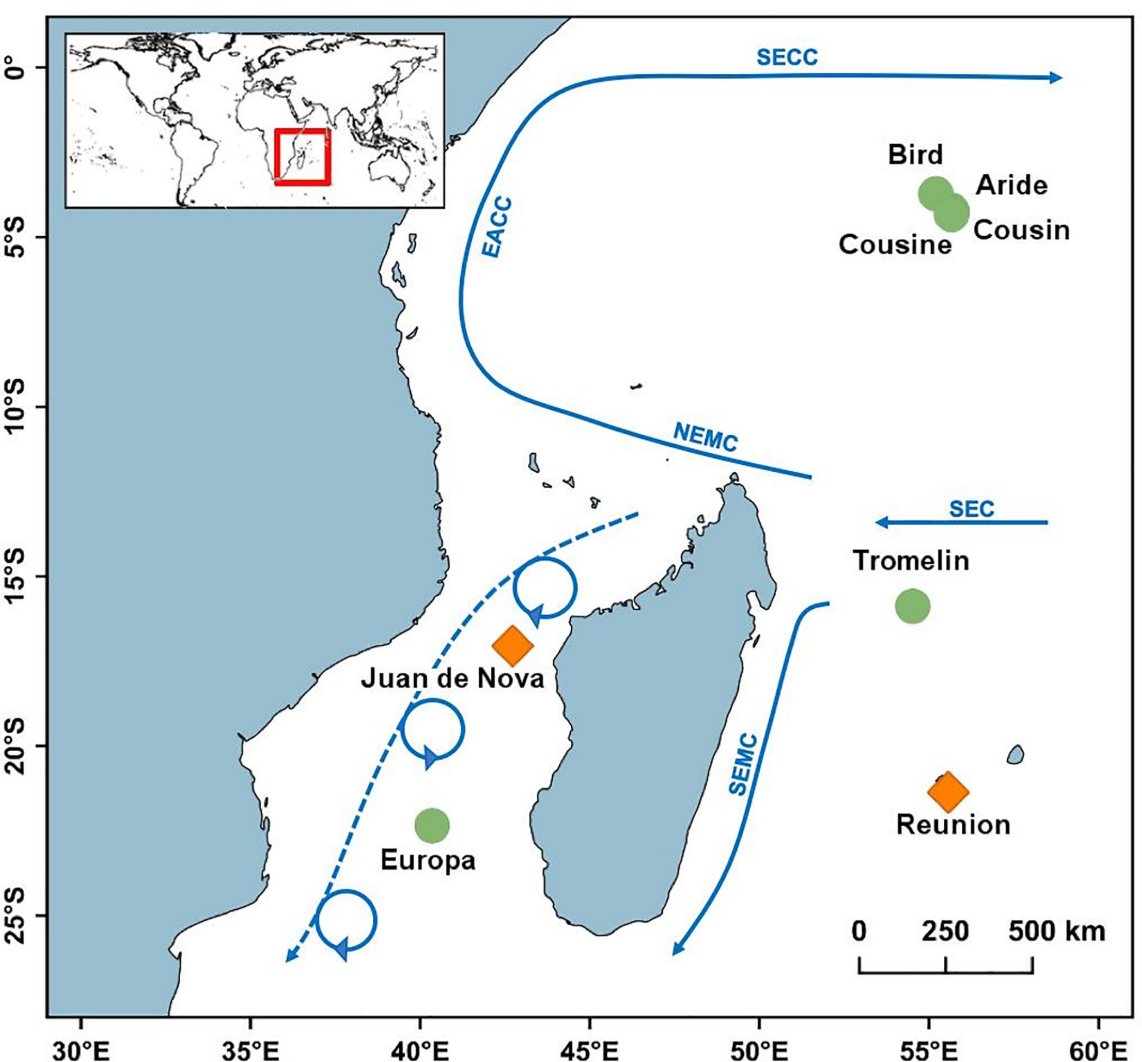
Location of the eight Western Indian Ocean islands where seabird populations were sampled for the detection of *Toxoplasma gondii* antibodies between 2011 and 2015. The orange lozenges correspond to islands inhabited by cats, the green dots to islands free of cats. Blue arrows indicated surface marine currents. SEC = South Equatorial Current, NEMC = North-East Madagascar Current, SEMC = South-East Madagascar Current, EACC = East African Coastal Current, SECC = South Equatorial Counter Current. Dashed arrow in the Mozambique Channel shows eddy circulation. Source: Schott et al. (2009). https://doi.org/10.1029/2007RG000245 [56].

Sampling was conducted on eight oceanic islands of the Western Indian Ocean (Fig 1): Reunion Island is part of the Mascarenes Archipelago; Aride, Bird, Cousin and Cousine are part of the Seychelles Archipelago; Tromelin lies between the Mascarenes Archipelago and the Seychelles Archipelago; Juan de Nova and Europa are in the Mozambique Channel.

The sampled islands have different histories regarding the presence of cats (Table 1). Cats were likely introduced to Reunion Island in the 17^th^ century and now occupy all habitats on the island [57]. Cats were introduced on Juan de Nova in the 20^th^ century and their population was significantly reduced between 2006 and 2011, but not eradicated at that time [58]. In the Seychelles Archipelago, cats used to be present on Bird [59, 60], Aride [61] and Cousine but were eradicated several decades ago [62]. Cousin, Europa and Tromelin have always been free of cats [62, 63]. In addition, Cousin, Cousine, Aride and Bird are approximately 2 km, 5 km, 9 km and 80 km from Praslin, the nearest cat-inhabited island of the Seychelles archipelago, while Europa and Tromelin are approximatively 300 km and 430 km away from the closest feline population (Table 1).

**Table 1:** Information on the 1014 seabirds sampled in the Western Indian Ocean between 2011 and 2015 whose sera were tested for the detection of *Toxoplasma gondii* antibodies (MAT ≥ 10).

Island	Presence/absence of felids	Species		Age-class	No tested	No positive	%
Aride 4°12’46”S, 55°39’53”E	Domestic cats used to be present but eradicated several decades ago. Approximately 9 km away from the nearest island inhabited by cats.	Wedge-tailed shearwater	*Ardenna pacifica*	Adults	8	0	0
		Sooty tern	*Onychoprion fuscatus*	Adults	33	12	36.4
Bird 3°53’S 55°12’E	Domestic cats used to be present but eradicated several decades ago. Approximately 80 km away from the nearest island inhabited by cats.	Brown noddy	*Anous stolidus*	Adults	51	9	17.6
		Lesser noddy	*Anous tenuirostris*	Adults	22	0	0
		Sooty tern	*Onychoprion fuscatus*	Adults	100	13	13.0
		White-tailed tropicbird	*Phaethon lepturus*	Adults	13	4	30.8
Cousin 4°19’S 55°39’E	Never colonized by felids Approximately 2 km away from the nearest island inhabited by cats	Brown noddy	*Anous stolidus*	Adults	29	13	44.8
		Lesser noddy	*Anous tenuirostris*	Adults	22	4	18.2
		Bridled tern	*Onychoprion anaethetus*	Adults	17	9	52.9
		White-tailed tropicbird	*Phaethon lepturus*	Adults	18	0	0
		Wedge-tailed shearwater	*Ardenna pacifica*	Adults	31	1	3.2
Cousine 4°21’S 55°38’E	Domestic cats used to be present but eradicated several decades ago. Approximately 5 km away from the nearest island inhabited by cats	Brown noddy	*Anous stolidus*	Adults	28	8	28.6
		Lesser noddy	*Anous tenuirostris*	Adults	31	1	3.2
		Wedge-tailed shearwater	*Ardenna pacifica*	Adults	24	0	0
Juan de Nova 17°03’S 42°45’E	Domestic cats introduced in the 20^th^. Population reduced between 2006 and 2011 but not eradicated at that time	Sooty tern	*Onychoprion fuscatus*	Adults	98	20	20.4
				Chicks	57	0	0
Reunion Island 21°22’S, 55°34’E	Domestic cats introduced in the 17^th^ and they occupy all habitats at that time	Wedge-tailed shearwater	*Ardenna pacifica*	Adults	50	5	10.0
				Chicks	23	1	4.3
Europa 22°20’S 40°22’E	Never colonized by felids. Approximately 300 km away from the closest feline population.	Great frigatebird	*Fregata minor*	Adults	14	0	0
				Chicks	22	0	0
		Red-footed booby	*Sula sula*	Adults	36	3	8.3
				Chicks	17	2	11.8
		Red-tailed tropicbird	*Phaethon rubricauda*	Adults	34	2	5.9
		Sooty tern	*Onychoprion fuscatus*	Adults	138	45	32.6
				Chicks	30	0	0
		White-tailed tropicbird	*Phaethon lepturus*	Adults	31	6	19.4
Tromelin 15°53’S 54°31’E	Never colonized by felids. Approximately 430 km away from the closest feline population.	Red-footed booby	*Sula sula*	Adults	8	0	0
				Chicks	10	1	10.0
		Masked booby	*Sula dactylatra*	Adults	5	2	40.0
				Juveniles	14	9	64.3
Total					1014	170	16.8

In total, 1014 individuals belonging to ten seabird species were included in this study (Table 1 and S1 Table). Most samples were collected between 2011 and 2013 as part of a previous study [64] except on Cousin and Cousine where samples were collected in 2015. The sampling strategy was designed to include a maximum number of species on each island. This sampling was adjusted in relation to local geographic, safety and ethical constraints that restrict access to bird colonies, such as in highly mountainous regions (*e*.*g*. Reunion Island) or for species highly sensitive to human disturbance (*e*.*g*. great frigatebird, *Fregata minor*). Birds were captured with bare hands or hand nets. Individual birds were categorized as chicks (non-flying birds fully dependent on parental feeding), juveniles (sexually immature flying birds), or adults (sexually mature birds, breeding or non-breeding). Whole blood (maximum of 1.0% of body weight) was collected from the medial metatarsal or basilic veins, as appropriate for each species. In the field, blood samples were collected in 2 ml micro-tubes placed in a cooler with ice packs and centrifuged within 12 hours after collection. Sera were transferred in cryogenic tubes and stored at -20°C. Samples were shipped to the laboratory in Reunion Island within a week and held at -20°C until tested.

### Serological assay

Sera were examined by the Modified agglutination test (MAT) described by Dubey and Desmonts [65]. This serological assay is the most sensitive, specific and used for the detection of IgG antibodies against *T*. *gondii* in birds [66, 67]. MAT antigen consisted of formalinized tachyzoïtes produced at the Laboratory of Parasitology, National Centre on Toxoplasmosis, Reims, France. Sera were first screened using 1:6, 1:10 and 1:25 dilutions in phosphate-buffered saline solution (PBS, pH 7.2). Those agglutinating the antigen at one (or more) of these screening dilutions were further tested in a serial 2-fold dilution, to a maximum dilution of 1:12800. Serum samples with agglutination at MAT titre ≥10 (i.e. serum dilution ≥ 1:25) were considered positive for the presence of *T*. *gondii* antibodies [67, 68]. Samples showing agglutination at further dilution were also mentioned to allow comparisons with literature data based on different dilution thresholds.

### Statistical analyses

Pearson Chi square test (χ^2^) were used to investigate the effect of the bird species, island, bird age-class (adult *versus* chick), and nest type (tree-nesting *vs* ground-nesting, S1 Table) on the probability of successful detection of *T*. *gondii* antibodies. Juveniles (N = 14) were excluded from the analysis because of the very low number of sampled birds as compared to chicks (N = 159) and adults (N = 841). Analyses were conducted in R 3.6.3 [69].

## Results

Antibodies against *T*. *gondii* were detected on all islands and all species, except the great frigatebird (Table 1). The overall seroprevalence was 16.8% [95% CI: 14.5%-19.1%]. MAT titres for the 170 seropositive birds ranged from 10 to 400 (Table 2).

**Table 2:** Number of samples tested positive for *Toxoplasma gondii* antibodies per species, age-class and titre of the Modified Agglutination Test (MAT). In brackets: corresponding dilution.

Species	Age class	MAT titre					
		≥ 10 (1:25)	≥ 25 (1:50)	≥ 50 (1:100)	≥ 100 (1:200)	≥ 200 (1:400)	≥ 400 (1:800)
Bridled tern *(Onychoprion anaethetus)*	Adult	9	6	0	0	0	0
Sooty tern *(Onychoprion fuscatus)*	Adult	90	56	31	9	3	1
Brown noddy *(Anous stolidus)*	Adult	30	13	3	1	0	0
Lesser noddy *(Anous tenuirostris)*	Adult	5	4	2	0	0	0
White-tailed tropicbird *(Phaethon lepturus)*	Adult	10	4	0	0	0	0
Red-tailed tropicbird *(Phaethon rubricauda)*	Adult	2	2	0	0	0	0
Masked booby *(Sula dactylatra)*	Adult	2	1	0	2	0	0
	Juvenile	9	9	3	0	1	0
Red-footed booby *(Sula sula)*	Adult	3	1	0	0	0	0
	Chick	3	1	0	2	0	0
Wedge-tailed shearwater *Ardenna pacifica*	Adult	6	2	2	0	0	0
	Chick	1	1	0	0	0	1
	**Total**	170	93	40	13	4	1

**Fig 2:**
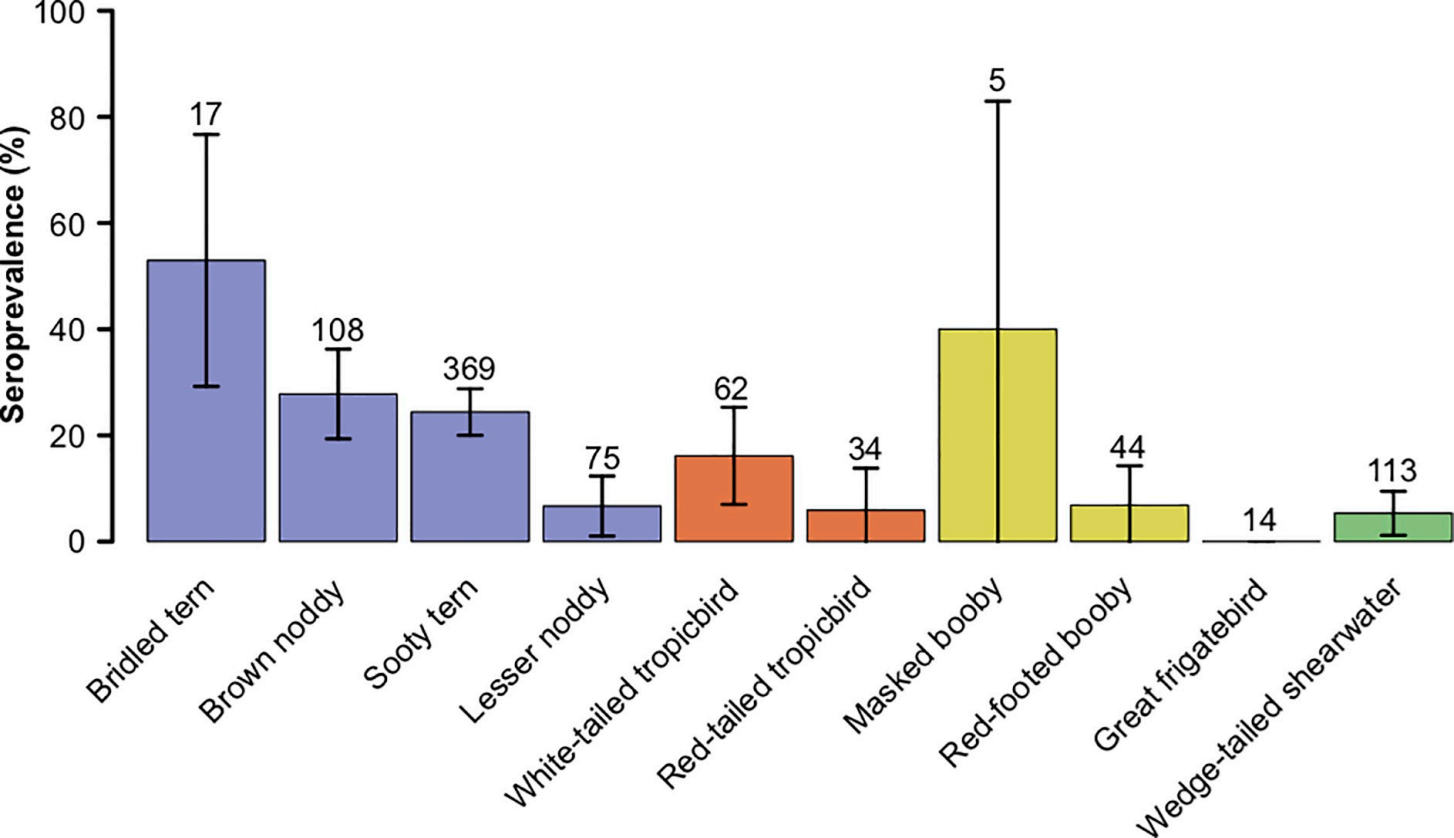
Seroprevalence of antibodies to *Toxoplasma gondii* per species in the adult seabirds sampled in the Western Indian Ocean (sample size and percentage with 95% confidence intervals). Colours indicate bird orders (blue: Charadriiformes, red: Phaethontiformes, yellow: Suliformes, green: Procellariformes).

The prevalence of *T*. *gondii* antibodies varied according to bird species (χ^2^ = 69, df = 990, *p* < 0.001), islands (χ^2^ = 17, df = 992, *p* < 0.05) and bird age class (χ^2^ = 36, df = 998, *p* < 0.001). However, differences between bird age classes should be interpreted cautiously because of the low number of chicks as compared to adults, and of the uneven distribution of the sampled chicks for each species (Table 1). The probability of detection of *T*. *gondii* antibodies varied between bird species in both chicks (χ^2^ = 10.3, df = 155, *p* < 0.05) and adults (χ^2^ = 66, df = 831, *p* < 0.001; Fig 2).

**Fig 3:**
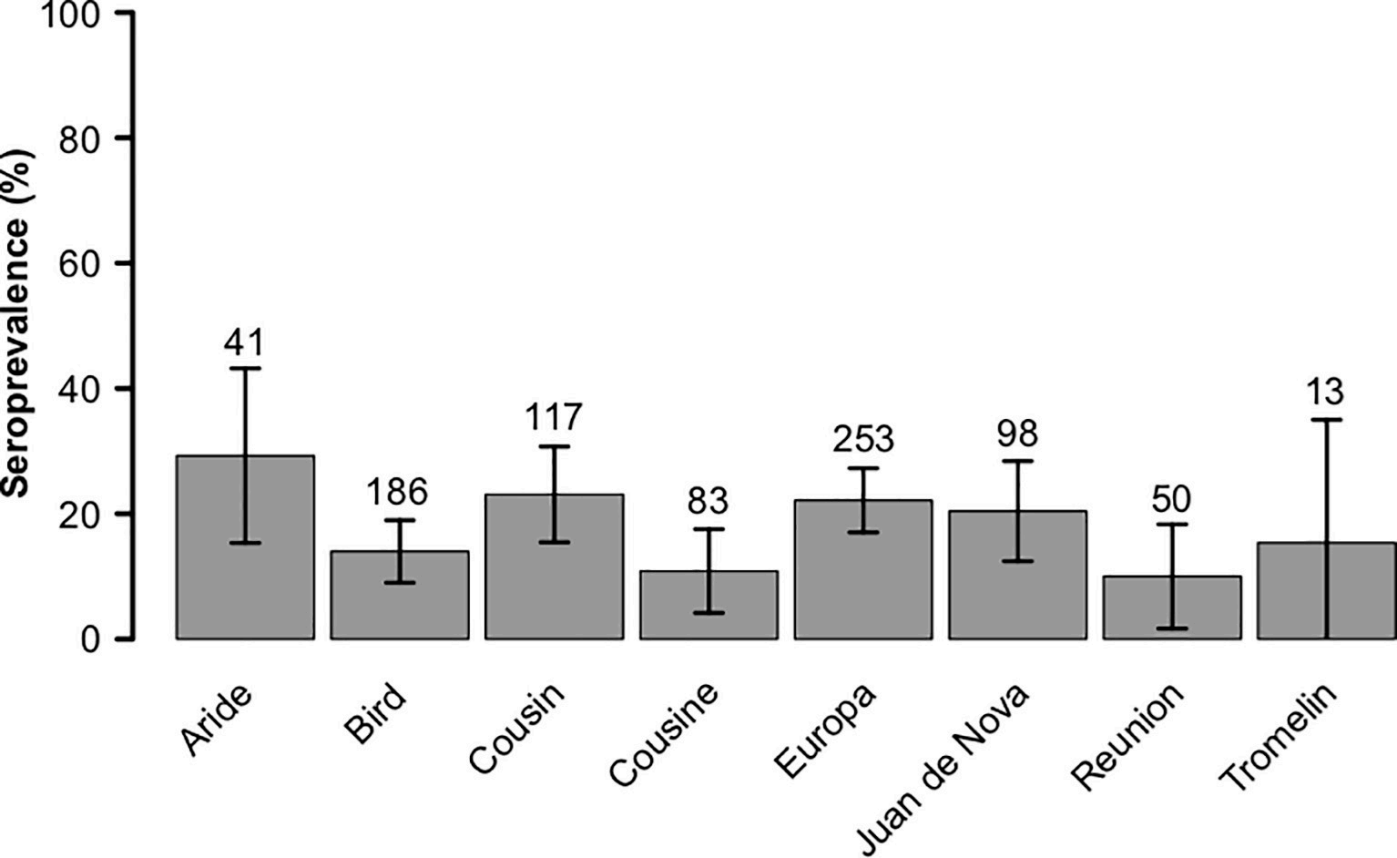
Seroprevalence of antibodies to *Toxoplasma gondii* per islands in the adult seabirds sampled in the Western Indian Ocean (sample size and percentage with 95% confidence intervals). Reunion and Juan de Nova are the only islands inhabited by cats.

In adults, *T*. *gondii* prevalence was 5.3% ± 4.1% in wedge-tailed shearwater, 5.8% ± 7.9% in red-tailed tropicbird, 6.7% ± 5.6% in lesser noddy, 6.8% ± 7.4% in red-footed booby, 24.4% ± 4.4% in sooty tern, 27.7% ± 8.4% in brown noddy, 40% ± 43% in masked booby, and 52.9% ± 23.7% in bridled tern. Prevalence of *T*. *gondii* in adults was significantly lower in tree-nesting than ground-nesting species (6% ± 4% *versus* 21% ± 3%, χ^2^ = 21, df = 839, *p* < 0.001). The probability of detection of *T*. *gondii* antibodies in adults varied significantly between islands (χ^2^ = 16, df = 833, *p* < 0.05; Fig 3) but prevalence on islands inhabited by cats (Reunion and Juan de Nova) did not significantly differ from prevalence on cat-free islands (χ^2^ = 0.38, df = 839, *p* < 0.53).

**Fig 4:**
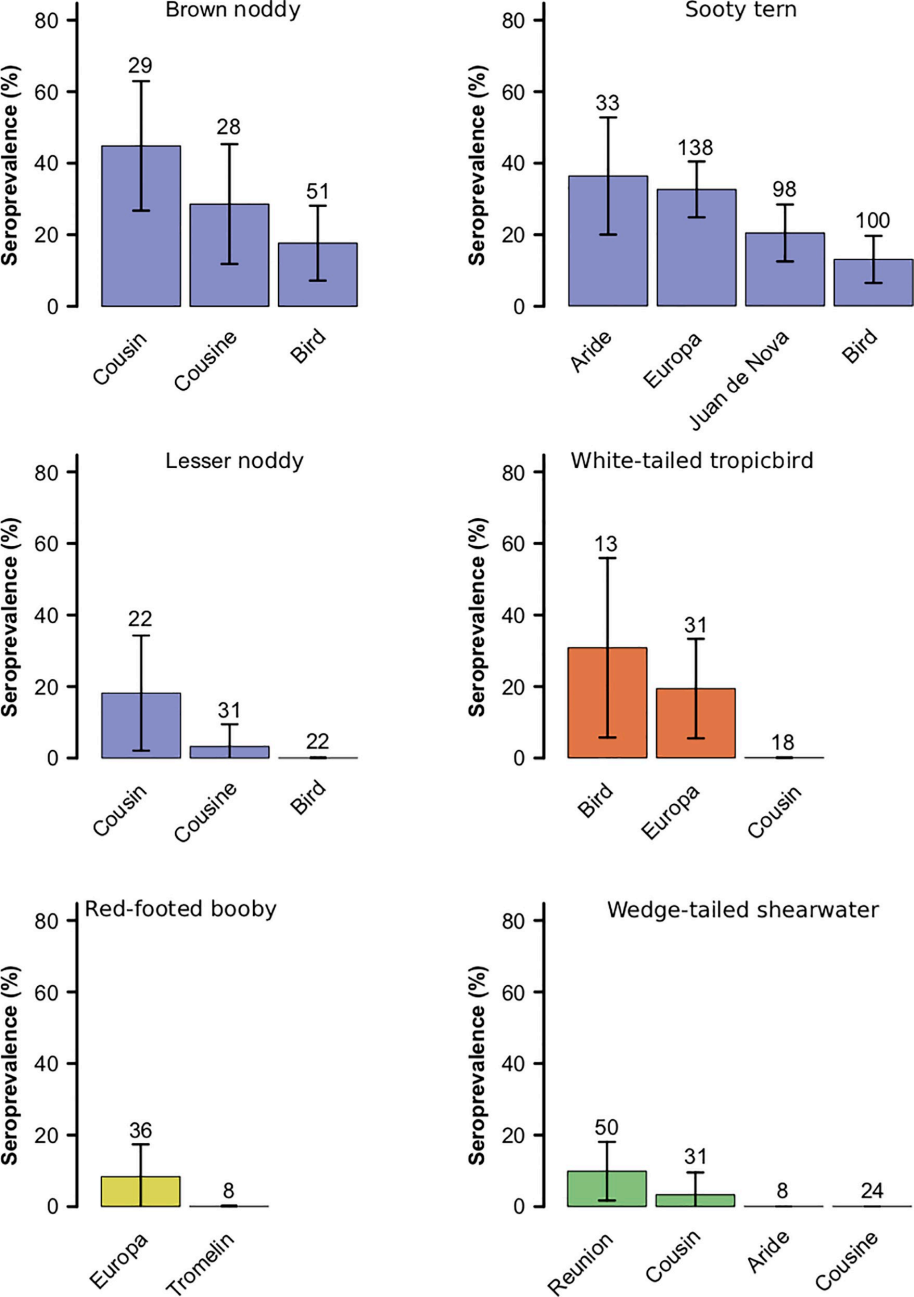
Seroprevalence of antibodies to *Toxoplasma gondii* per species and island in the adult seabirds sampled in the Western Indian Ocean (sample size and percentage with 95% confidence intervals). Sample sizes are indicated above bars. Colours indicate bird orders (blue: Charadriiformes, red: Phaethontiformes, yellow: Suliformes, green: Procellariformes). Juan de Nova and Reunion are inhabited by cats; Aride, Cousin and Cousine are less than 10 km away from the nearest island inhabited by cats; Bird, Europa and Tromelin are 80 km to 430 km away from the nearest feline population.

Differences in the prevalence of *T*. *gondii* in adults were also detected between populations (*i*.*e*. islands) of the same species (Fig 4) in brown noddy (χ^2^ = 6.7, df = 105, *p* < 0.05), sooty tern (χ^2^ = 16, df = 365, *p* < 0.001), lesser noddy (χ^2^ = 7.1, df = 72, *p* < 0.05) and white-tailed tropicbird (χ^2^ = 8.2, df = 59, *p* < 0.05) but not in red-footed booby (χ^2^ = 1.3, df = 42, *p* = 0.26) and wedge-tailed shearwater (χ^2^ = 5.6, df = 109, *p* = 0.14).

## Discussion

Based on the analysis of 1014 seabirds belonging to ten species sampled in the Western Indian Ocean, we found an overall prevalence of 16.8% of seabirds carrying antibodies against *T*. *gondii*. This prevalence was higher than the one reported with the same threshold in the masked booby, the brown booby (*Sula leucogaster*) and the red-billed tropicbird (*Phaethon aethereus*) sampled in the Abrolhos archipelago, the south of Bahia State (Brazil) in the Atlantic Ocean (5.8% at MAT titre ≥ 10) [53]. If we had considered only MAT titres ≥ 25, *T*. *gondii* prevalence would have been of 9.17% (93/1014), thus also higher than prevalence reported for other seabird species such as the Galapagos penguin (*Spheniscus mendiculus*) and the flightless cormorant (*Phalacrocorax harrisi*), both sampled in the Galapagos Archipelago (Ecuador) in the Pacific Ocean (2.3% at MAT titre ≥ 25) [54]. In birds, clinical signs associated with toxoplasmosis include anorexia, diarrhoea and respiratory distress, and may occasionally result in death [24, 25, 66]. Fatal toxoplasmosis has notably been reported in captive penguins [70, 71] and in a free-ranging red-footed booby died of disseminated toxoplasmosis on a Hawaiian island [72]. The relatively high exposure to *T*. *gondii* in the Western Indian Ocean therefore raises questions about the risk of induced mortality in seabird populations, although all birds sampled in this study were apparently healthy. Further investigations could be performed to detect clinical toxoplasmosis on these populations from necropsies and molecular analysis conducted on freshly dead birds, in particular in colonies with high *T*. *gondii* prevalence and/or in species susceptible to this infection, such as the red-footed booby.

As expected, the prevalence of *T*. *gondii* in seabirds sampled in the Western Indian Ocean varied significantly by age class, species and island, and was higher in ground nesting birds than tree-nesting birds confirming that ground-contact is a risk factor for seabirds to *T*. *gondii*. Of the species we sampled, the wedge-tailed shearwater has the most contact with the ground as it nests in a burrow [73]. On Reunion Island, wedge-tailed shearwaters burrow in cliffs frequented by cats. Those that tested positive for *T*. *gondii* antibodies had most likely ingested oocysts while preening their feathers stained with oocyst-contaminated soil. For sooty terns, the prevalence of anti-*T*. *gondii* antibodies was higher on Aride (where cats were eradicated several decades ago) than on Juan de Nova (where cats were present at the time of our sampling) raising the question of how long *T*. *gondii* oocysts can persist in the environment after cat eradication. Experimentally, the proportion of oocysts surviving in soil after 100 days is around 7% under dry conditions and 44% under damp conditions [17]. In Baja California, Mexico, the rate of recent human exposure to *T*. *gondii* (estimated via IgM detection) was 12–26% on five islands inhabited by cats and only 1.8% on the island where cats were eradicated seven years earlier [74]. The persistence of infectious oocysts for decades after eradication of the cats on Aride therefore seems unlikely. This implies that seabirds testing positive for *T*. *gondii* antibodies on Aride as well as Cousine and Bird (where cats were also eradicated several decades ago) were necessarily exposed to oocysts that were not produced locally and therefore dispersed from their shedding site. The medium to long-range dispersal of oocysts from land or islands inhabited by felids may also explain why we did not find a higher prevalence of *T*. *gondii* in seabirds on islands inhabited by cats than on cat-free islands.

Our data broadly suggest that birds visiting the shore are the most exposed to *T*. *gondii*. Indeed, the highest seroprevalence was observed in bridled terns, sooty terns and brown noddies (Charadriiformes) which nest close to the sea. In New Caledonia, bridled terns nest at less than five meters above the high tide level and at less than eight meters away from the water mark [75]. In the Seychelles, sooty terns nest on open sand or on sand with scattered low vegetation above the high tide level [76, 77]. Similarly, brown noddies nest both on the ground and in trees and often rest and collect nest material on the ground and on shores. Lesser noddies only nest in trees but spend time on the ground and shore, sunbathing during the day and collecting soil-borne materials for nesting (e.g. sticks and leaves) as well as material floating on the sea. In the Seychelles, the prevalence of *T*. *gondii* in sooty tern and noddy populations decreased with distance from the nearest cat population: for brown and lesser noddies, it was significantly higher on Cousin and Cousine islands (2 km and 5 km from Praslin) than on Bird island (80 km from Praslin); For sooty tern, it was higher on Aride island (9 km from Praslin) than on Bird. This pattern of *T*. *gondii* prevalence decrease with distance to cat populations was not observed for the white-tailed tropicbird, a more inland species than sooty tern and noddies. Taken together, these observations advise that *T*. *gondii* oocysts produced on cat-inhabited land could be transported by oceanic currents and deposited on distant shorelines, thereby contributing to the exposure of birds exploiting these habitats, such as terns and noddies. Shapiro et al. [40] suggested that the attachment of *T*. *gondii* oocysts to marine aggregates may significantly influence the water transport of this terrestrial parasite. This association of oocysts with marine aggregates may also presumably facilitate their transport from islands colonised by cats. On arrival at distant shores, oocysts may be retained by high-water mark since they adhere to kelp [78]. The habit of noddies to collect seaweed for incorporation into their nests could prolong their exposure to *T*. *gondii*.

However, the detection of *T*. *gondii* antibodies in species that usually do not spend time in coastal habitats (tropicbirds, shearwater and boobies) suggests that a third source of contamination could also be involved in the transmission route of *T*. *gondii* to pelagic seabirds. Infectious *T*. *gondii* oocysts and/or *T*. *gondii* DNA have been detected in the intestines or tissues of Mullidae (goatfish), Carangidae (trevally, mackerel), Engraulidae (anchovies) and Clupeidae (herrings, shads, sardines) [43, 79]. Clupidae, Carangidae and Clupidae fishes are preyed by *Tursiop truncatus* and *Delphinus delphis* [80, 81] which are the two dolphin species most exposed to *T*. *gondii* in the Mediterranean Sea [46]. In the Seychelles, Carangidae and Engraulidae fishes are the secondary prey of white-tailed tropicbirds [82]. Similarly, on Europa, the red-tailed tropicbird and the red-footed booby occasionally take Carangidae and/or Mullidae fish [83, 84]. Therefore, the few white-tailed tropicbirds that tested positive on Bird and Europa, as well as the red-tailed tropicbirds and red-footed boobies that tested positive on Europa, may have been exposed to *T*. *gondii* by feeding on Carangidae or Mullidae fish carrying infectious oocysts. In the same way, the high *T*. *gondii* prevalence in sooty terns and brown noddies sampled in the Seychelles and the Mozambique Channel may not only result from their use of the shore but also to the significance of Mullidae and Carangidae in their diet, which also occasionally includes Clupeidae and Engraulidae [82, 83, 85]. Interestingly, comparable prevalence of *T*. *gondii* were detected in the Aride and Europa sooty tern populations (36.4% and 32.6%) which also have major similarities in diet composition [86]. Taken together, these observations suggest that Mullidae and Carangidae, and possibly Clupeidae and Engraulidae, may serve as biotic carriers for *T*. *gondii* in the Western Indian Ocean.

As expected, prevalence of *T*. *gondii* antibodies was lower in chicks than on adults in sooty tern (Juan de Nova: 0% *versus* 20.4%; Europa: 0% *versus* 32.6%) and in wedge-tailed shearwaters (Reunion: 4.3% *versus* 10%). However, prevalence of *T*. *gondii* was higher in chicks than in adults in red-footed boobies sampled on Europa (11.8% *versus* 8.3%) and Tromelin (1/1 positive *versus* 0/8). This unexpected result can be due to the persistence of maternal antibodies transferred via egg yolk [87, 88]. In long-lived birds such as wedge-tailed shearwater or red-footed boobies, specific maternal antibodies can have an estimated half-life of 25 days post-hatching [89, 90]. The low antibody levels detected in one shearwater and three red-footed booby chicks (MAT titres = 10 or 25) most likely resulted from maternal antibody transfer since antibody level might have been higher if chicks had produced antibodies in response to a recent environmental exposure to *T*. *gondii*. In contrast, the high antibody levels detected in nine juvenile masked boobies (MAT titres = 50, 100 or 200) from Tromelin, located 430 km away from the closest feline population, as well as in adult red-footed and masked boobies on Europa and Tromelin (300 km and 430 km away from the closest feline population) likely resulted of an environmental exposure to *T*. *gondii*. This result is intriguing because adult masked and red-footed boobies have a foraging range limited to the 150 km surrounding Europa and Tromelin [91–93]. The detection of antibodies to *T*. *gondii* in boobies from these islands could a result of the long-distance movements that juvenile boobies sometimes make before breeding [94–96] and/or the transport of oocysts across the ocean for hundreds of kilometres.

To conclude, this study clearly demonstrates that *T*. *gondii* has efficiently colonized the marine realm of the tropical Indian Ocean. Three non-exclusive routes of contamination could be involved: (i) by the ingestion of oocysts locally deposited on islands colonised by cats; (ii) by the ingestion of oocysts transported by currents and deposited on the shore of distant islands; (iii) by the ingestion of oocysts carried by Mullidae, Carangidae, Clupeidae or Engraulidae fish. It is interesting to note that the only species for which no seropositive bird was found—i.e. great frigatebird breeding on Europa—was also the least exposed to these routes of contamination. Indeed, on the cat-free island of Europa, great frigatebirds nest and roost in trees and bushes and have a diet essentially composed of flying-fish and Ommastrephid squids [55, 97]. Further investigations are needed to confirm that *T*. *gondii* oocysts could be transported over tens or hundreds of kilometres across the ocean and to better identify the ecological processes allowing the pathway of this protozoa in the tropical seabird community.

## Supporting information

S1 Table Information on seabirds sampled in the western Indian Ocean for the detection of antibodies against Toxoplasma gondii in their sera. (PDF)
