# Supplementary material for: Exposure of pelagic seabirds to Toxoplasma gondii in the Western Indian Ocean points to an open sea dispersal of this terrestrial parasite
Source: PLoS One. 2021 Aug 18;16(8):e0255664. doi: 10.1371/journal.pone.0255664 (PMC8372946; doi:10.1371/journal.pone.0255664)
Supplement: S1 Table — (PDF) [file pone.0255664.s001.pdf]

ID	Species	Island	Year	Nest	T. gondii antibodies
FS34516	*Ardenna_pacifica*	Aride	2012	ground_nester	0
FS34530	*Ardenna_pacifica*	Aride	2012	ground_nester	0
FS34531	*Ardenna_pacifica*	Aride	2012	ground_nester	0
FS34533	*Ardenna_pacifica*	Aride	2012	ground_nester	0
FS34539	*Ardenna_pacifica*	Aride	2012	ground_nester	0
FS34540	*Ardenna_pacifica*	Aride	2012	ground_nester	0
FS34541	*Ardenna_pacifica*	Aride	2012	ground_nester	0
GY152219	*Ardenna_pacifica*	Aride	2012	ground_nester	0
ONYFUS020	*Onychoprion_fuscatus*	Aride	2012	ground_nester	0
ONYFUS026	*Onychoprion_fuscatus*	Aride	2012	ground_nester	0
ONYFUS030	*Onychoprion_fuscatus*	Aride	2012	ground_nester	0
ONYFUS033	*Onychoprion_fuscatus*	Aride	2012	ground_nester	0
ONYFUS039	*Onychoprion_fuscatus*	Aride	2012	ground_nester	0
ONYFUS041	*Onychoprion_fuscatus*	Aride	2012	ground_nester	0
ONYFUS043	*Onychoprion_fuscatus*	Aride	2012	ground_nester	0
ONYFUS048	*Onychoprion_fuscatus*	Aride	2012	ground_nester	0
ONYFUS054	*Onychoprion_fuscatus*	Aride	2012	ground_nester	0
ONYFUS057	*Onychoprion_fuscatus*	Aride	2012	ground_nester	1
ONYFUS060	*Onychoprion_fuscatus*	Aride	2012	ground_nester	0
ONYFUS061	*Onychoprion_fuscatus*	Aride	2012	ground_nester	0
ONYFUS065	*Onychoprion_fuscatus*	Aride	2012	ground_nester	0
ONYFUS067	*Onychoprion_fuscatus*	Aride	2012	ground_nester	0
ONYFUS071	*Onychoprion_fuscatus*	Aride	2012	ground_nester	0
ONYFUS072	*Onychoprion_fuscatus*	Aride	2012	ground_nester	0
ONYFUS073	*Onychoprion_fuscatus*	Aride	2012	ground_nester	0
ONYFUS076	*Onychoprion_fuscatus*	Aride	2012	ground_nester	1
ONYFUS081	*Onychoprion_fuscatus*	Aride	2012	ground_nester	0
ONYFUS082	*Onychoprion_fuscatus*	Aride	2012	ground_nester	1
ONYFUS084	*Onychoprion_fuscatus*	Aride	2012	ground_nester	0
ONYFUS085	*Onychoprion_fuscatus*	Aride	2012	ground_nester	1
ONYFUS086	*Onychoprion_fuscatus*	Aride	2012	ground_nester	0
ONYFUS087	*Onychoprion_fuscatus*	Aride	2012	ground_nester	0
ONYFUS089	*Onychoprion_fuscatus*	Aride	2012	ground_nester	1
ONYFUS090	*Onychoprion_fuscatus*	Aride	2012	ground_nester	1
ONYFUS091	*Onychoprion_fuscatus*	Aride	2012	ground_nester	1
ONYFUS092	*Onychoprion_fuscatus*	Aride	2012	ground_nester	1
ONYFUS093	*Onychoprion_fuscatus*	Aride	2012	ground_nester	1

ONYFUS096	*Onychoprion_fuscatus*	Aride	2012	ground_nester	1
ONYFUS098	*Onychoprion_fuscatus*	Aride	2012	ground_nester	1
ONYFUS100	*Onychoprion_fuscatus*	Aride	2012	ground_nester	0
ONYFUS101	*Onychoprion_fuscatus*	Aride	2012	ground_nester	1
BI-BN008	*Anous_stolidus*	Bird	2012	ground_nester	1
BI-BN011	*Anous_stolidus*	Bird	2012	ground_nester	0
BI-BN014	*Anous_stolidus*	Bird	2012	ground_nester	0
BI-BN028	*Anous_stolidus*	Bird	2012	ground_nester	0
BI-BN033	*Anous_stolidus*	Bird	2012	ground_nester	0
BI-BN035	*Anous_stolidus*	Bird	2012	ground_nester	0
BI-BN036	*Anous_stolidus*	Bird	2013	ground_nester	1
BI-BN038	*Anous_stolidus*	Bird	2013	ground_nester	0
BI-BN039	*Anous_stolidus*	Bird	2013	ground_nester	0
BI-BN040	*Anous_stolidus*	Bird	2013	ground_nester	0
BI-BN042	*Anous_stolidus*	Bird	2013	ground_nester	0
BI-BN044	*Anous_stolidus*	Bird	2013	ground_nester	0
BI-BN045	*Anous_stolidus*	Bird	2013	ground_nester	0
BI-BN048	*Anous_stolidus*	Bird	2013	ground_nester	0
BI-BN049	*Anous_stolidus*	Bird	2013	ground_nester	0
BI-BN052	*Anous_stolidus*	Bird	2013	ground_nester	0
BI-BN054	*Anous_stolidus*	Bird	2013	ground_nester	0
BI-BN057	*Anous_stolidus*	Bird	2013	ground_nester	0
BI-BN058	*Anous_stolidus*	Bird	2013	ground_nester	0
BI-BN060	*Anous_stolidus*	Bird	2013	ground_nester	0
BI-BN061	*Anous_stolidus*	Bird	2013	ground_nester	0
BI-BN062	*Anous_stolidus*	Bird	2013	ground_nester	0
BI-BN070	*Anous_stolidus*	Bird	2013	ground_nester	1
BI-BN072	*Anous_stolidus*	Bird	2013	ground_nester	1
BI-BN076	*Anous_stolidus*	Bird	2013	ground_nester	0
BI-BN077	*Anous_stolidus*	Bird	2013	ground_nester	0
BI-BN079	*Anous_stolidus*	Bird	2013	ground_nester	0
BI-BN083	*Anous_stolidus*	Bird	2013	ground_nester	0
BI-BN084	*Anous_stolidus*	Bird	2013	ground_nester	0
BI-BN087	*Anous_stolidus*	Bird	2013	ground_nester	1
BI-BN088	*Anous_stolidus*	Bird	2013	ground_nester	0
BI-BN089	*Anous_stolidus*	Bird	2013	ground_nester	0
BI-BN090	*Anous_stolidus*	Bird	2013	ground_nester	0
BI-BN091	*Anous_stolidus*	Bird	2013	ground_nester	0
BI-BN092	*Anous_stolidus*	Bird	2013	ground_nester	0

BI-BN094	*Anous_stolidus*	Bird	2013	ground_nester	0
BI-BN099	*Anous_stolidus*	Bird	2013	ground_nester	1
BI-BN100	*Anous_stolidus*	Bird	2013	ground_nester	0
BI-BN101	*Anous_stolidus*	Bird	2013	ground_nester	0
BI-BN104	*Anous_stolidus*	Bird	2013	ground_nester	0
BI-BN105	*Anous_stolidus*	Bird	2013	ground_nester	0
BI-BN106	*Anous_stolidus*	Bird	2013	ground_nester	0
BI-BN107	*Anous_stolidus*	Bird	2013	ground_nester	1
BI-BN108	*Anous_stolidus*	Bird	2013	ground_nester	0
BI-BN110	*Anous_stolidus*	Bird	2013	ground_nester	1
BI-BN115	*Anous_stolidus*	Bird	2013	ground_nester	0
BI-BN117	*Anous_stolidus*	Bird	2013	ground_nester	1
BI-BN118	*Anous_stolidus*	Bird	2013	ground_nester	0
BI-BN120	*Anous_stolidus*	Bird	2013	ground_nester	0
BI-BN123	*Anous_stolidus*	Bird	2013	ground_nester	0
BI-BN124	*Anous_stolidus*	Bird	2013	ground_nester	0
BI-LN065	*Anous_tenuirostris*	Bird	2013	tree-nesters	0
BI-LN072	*Anous_tenuirostris*	Bird	2013	tree-nesters	0
BI-LN077	*Anous_tenuirostris*	Bird	2013	tree-nesters	0
BI-LN079	*Anous_tenuirostris*	Bird	2013	tree-nesters	0
BI-LN081	*Anous_tenuirostris*	Bird	2013	tree-nesters	0
BI-LN087	*Anous_tenuirostris*	Bird	2013	tree-nesters	0
BI-LN088	*Anous_tenuirostris*	Bird	2013	tree-nesters	0
BI-LN090	*Anous_tenuirostris*	Bird	2013	tree-nesters	0
BI-LN099	*Anous_tenuirostris*	Bird	2013	tree-nesters	0
BI-LN103	*Anous_tenuirostris*	Bird	2013	tree-nesters	0
BI-LN104	*Anous_tenuirostris*	Bird	2013	tree-nesters	0
BI-LN106	*Anous_tenuirostris*	Bird	2013	tree-nesters	0
BI-LN113	*Anous_tenuirostris*	Bird	2013	tree-nesters	0
BI-LN119	*Anous_tenuirostris*	Bird	2013	tree-nesters	0
BI-LN123	*Anous_tenuirostris*	Bird	2013	tree-nesters	0
BI-LN124	*Anous_tenuirostris*	Bird	2013	tree-nesters	0
BI-LN129	*Anous_tenuirostris*	Bird	2013	tree-nesters	0
BI-LN136	*Anous_tenuirostris*	Bird	2013	tree-nesters	0
BI-LN142	*Anous_tenuirostris*	Bird	2013	tree-nesters	0
BI-LN143	*Anous_tenuirostris*	Bird	2013	tree-nesters	0
BI-LN147	*Anous_tenuirostris*	Bird	2013	tree-nesters	0
BI-LN152	*Anous_tenuirostris*	Bird	2013	tree-nesters	0
BI-ST001	*Onychoprion_fuscatus*	Bird	2012	ground_nester	0

BI-ST003	*Onychoprion_fuscatus*	Bird	2012	ground_nester	0
BI-ST011	*Onychoprion_fuscatus*	Bird	2012	ground_nester	0
BI-ST013	*Onychoprion_fuscatus*	Bird	2012	ground_nester	0
BI-ST014	*Onychoprion_fuscatus*	Bird	2012	ground_nester	0
BI-ST017	*Onychoprion_fuscatus*	Bird	2012	ground_nester	0
BI-ST023	*Onychoprion_fuscatus*	Bird	2012	ground_nester	0
BI-ST038	*Onychoprion_fuscatus*	Bird	2012	ground_nester	1
BI-ST042	*Onychoprion_fuscatus*	Bird	2012	ground_nester	0
BI-ST043	*Onychoprion_fuscatus*	Bird	2012	ground_nester	0
BI-ST047	*Onychoprion_fuscatus*	Bird	2012	ground_nester	0
BI-ST059	*Onychoprion_fuscatus*	Bird	2012	ground_nester	0
BI-ST060	*Onychoprion_fuscatus*	Bird	2012	ground_nester	0
BI-ST063	*Onychoprion_fuscatus*	Bird	2012	ground_nester	0
BI-ST064	*Onychoprion_fuscatus*	Bird	2012	ground_nester	0
BI-ST069	*Onychoprion_fuscatus*	Bird	2012	ground_nester	1
BI-ST070	*Onychoprion_fuscatus*	Bird	2012	ground_nester	0
BI-ST071	*Onychoprion_fuscatus*	Bird	2012	ground_nester	0
BI-ST073	*Onychoprion_fuscatus*	Bird	2012	ground_nester	0
BI-ST075	*Onychoprion_fuscatus*	Bird	2012	ground_nester	0
BI-ST076	*Onychoprion_fuscatus*	Bird	2012	ground_nester	0
BI-ST078	*Onychoprion_fuscatus*	Bird	2012	ground_nester	0
BI-ST081	*Onychoprion_fuscatus*	Bird	2012	ground_nester	0
BI-ST084	*Onychoprion_fuscatus*	Bird	2012	ground_nester	1
BI-ST085	*Onychoprion_fuscatus*	Bird	2012	ground_nester	0
BI-ST087	*Onychoprion_fuscatus*	Bird	2012	ground_nester	0
BI-ST088	*Onychoprion_fuscatus*	Bird	2012	ground_nester	0
BI-ST089	*Onychoprion_fuscatus*	Bird	2012	ground_nester	0
BI-ST090	*Onychoprion_fuscatus*	Bird	2012	ground_nester	0
BI-ST092	*Onychoprion_fuscatus*	Bird	2012	ground_nester	0
BI-ST093	*Onychoprion_fuscatus*	Bird	2012	ground_nester	1
BI-ST094	*Onychoprion_fuscatus*	Bird	2012	ground_nester	0
BI-ST095	*Onychoprion_fuscatus*	Bird	2012	ground_nester	0
BI-ST097	*Onychoprion_fuscatus*	Bird	2012	ground_nester	0
BI-ST098	*Onychoprion_fuscatus*	Bird	2012	ground_nester	0
BI-ST103	*Onychoprion_fuscatus*	Bird	2012	ground_nester	0
BI-ST104	*Onychoprion_fuscatus*	Bird	2012	ground_nester	0
BI-ST105	*Onychoprion_fuscatus*	Bird	2012	ground_nester	0
BI-ST106	*Onychoprion_fuscatus*	Bird	2012	ground_nester	0
BI-ST107	*Onychoprion_fuscatus*	Bird	2012	ground_nester	0

BI-ST108	Onychoprion_fuscatus	Bird	2012	ground_nester	0
BI-ST109	Onychoprion_fuscatus	Bird	2012	ground_nester	0
BI-ST111	Onychoprion_fuscatus	Bird	2012	ground_nester	0
BI-ST112	Onychoprion_fuscatus	Bird	2012	ground_nester	0
BI-ST113	Onychoprion_fuscatus	Bird	2012	ground_nester	0
BI-ST115	Onychoprion_fuscatus	Bird	2012	ground_nester	0
BI-ST117	Onychoprion_fuscatus	Bird	2012	ground_nester	0
BI-ST118	Onychoprion_fuscatus	Bird	2012	ground_nester	0
BI-ST120	Onychoprion_fuscatus	Bird	2012	ground_nester	0
BI-ST122	Onychoprion_fuscatus	Bird	2012	ground_nester	0
BI-ST124	Onychoprion_fuscatus	Bird	2012	ground_nester	0
BI-ST125	Onychoprion_fuscatus	Bird	2012	ground_nester	0
BI-ST126	Onychoprion_fuscatus	Bird	2012	ground_nester	0
BI-ST127	Onychoprion_fuscatus	Bird	2012	ground_nester	0
BI-ST128	Onychoprion_fuscatus	Bird	2012	ground_nester	0
BI-ST129	Onychoprion_fuscatus	Bird	2012	ground_nester	0
BI-ST130	Onychoprion_fuscatus	Bird	2012	ground_nester	0
BI-ST131	Onychoprion_fuscatus	Bird	2012	ground_nester	0
BI-ST135	Onychoprion_fuscatus	Bird	2012	ground_nester	1
BI-ST137	Onychoprion_fuscatus	Bird	2012	ground_nester	1
BI-ST140	Onychoprion_fuscatus	Bird	2012	ground_nester	0
BI-ST141	Onychoprion_fuscatus	Bird	2012	ground_nester	1
BI-ST143	Onychoprion_fuscatus	Bird	2012	ground_nester	0
BI-ST145	Onychoprion_fuscatus	Bird	2012	ground_nester	0
BI-ST146	Onychoprion_fuscatus	Bird	2012	ground_nester	0
BI-ST149	Onychoprion_fuscatus	Bird	2012	ground_nester	0
BI-ST150	Onychoprion_fuscatus	Bird	2012	ground_nester	0
BI-ST152	Onychoprion_fuscatus	Bird	2012	ground_nester	0
BI-ST153	Onychoprion_fuscatus	Bird	2012	ground_nester	0
BI-ST154	Onychoprion_fuscatus	Bird	2012	ground_nester	1
BI-ST156	Onychoprion_fuscatus	Bird	2012	ground_nester	0
BI-ST160	Onychoprion_fuscatus	Bird	2012	ground_nester	0
BI-ST162	Onychoprion_fuscatus	Bird	2012	ground_nester	0
BI-ST165	Onychoprion_fuscatus	Bird	2012	ground_nester	0
BI-ST166	Onychoprion_fuscatus	Bird	2012	ground_nester	0
BI-ST168	Onychoprion_fuscatus	Bird	2012	ground_nester	0
BI-ST170	Onychoprion_fuscatus	Bird	2012	ground_nester	0
BI-ST171	Onychoprion_fuscatus	Bird	2012	ground_nester	0
BI-ST173	Onychoprion_fuscatus	Bird	2012	ground_nester	0

BI-ST175	*Onychoprion_fuscatus*	Bird	2012	ground_nester	1
BI-ST176	*Onychoprion_fuscatus*	Bird	2012	ground_nester	1
BI-ST177	*Onychoprion_fuscatus*	Bird	2012	ground_nester	0
BI-ST178	*Onychoprion_fuscatus*	Bird	2012	ground_nester	0
BI-ST187	*Onychoprion_fuscatus*	Bird	2012	ground_nester	0
BI-ST191	*Onychoprion_fuscatus*	Bird	2012	ground_nester	0
BI-ST196	*Onychoprion_fuscatus*	Bird	2012	ground_nester	0
BI-ST203	*Onychoprion_fuscatus*	Bird	2012	ground_nester	0
BI-ST210	*Onychoprion_fuscatus*	Bird	2012	ground_nester	1
BI-ST211	*Onychoprion_fuscatus*	Bird	2012	ground_nester	0
BI-ST218	*Onychoprion_fuscatus*	Bird	2012	ground_nester	0
BI-ST219	*Onychoprion_fuscatus*	Bird	2012	ground_nester	0
BI-ST221	*Onychoprion_fuscatus*	Bird	2012	ground_nester	0
BI-ST222	*Onychoprion_fuscatus*	Bird	2012	ground_nester	0
BI-ST224	*Onychoprion_fuscatus*	Bird	2012	ground_nester	0
BI-ST227	*Onychoprion_fuscatus*	Bird	2012	ground_nester	0
BI-ST320	*Onychoprion_fuscatus*	Bird	2012	ground_nester	0
BI-ST322	*Onychoprion_fuscatus*	Bird	2012	ground_nester	0
BI-ST323	*Onychoprion_fuscatus*	Bird	2012	ground_nester	1
BI-ST325	*Onychoprion_fuscatus*	Bird	2012	ground_nester	0
BI-ST327	*Onychoprion_fuscatus*	Bird	2012	ground_nester	1
BI-WT05	*Phaethon_lepturus*	Bird	2012	ground_nester	0
BI-WT06	*Phaethon_lepturus*	Bird	2012	ground_nester	0
BI-WT14	*Phaethon_lepturus*	Bird	2012	ground_nester	1
BI-WT15	*Phaethon_lepturus*	Bird	2012	ground_nester	0
BI-WT16	*Phaethon_lepturus*	Bird	2012	ground_nester	1
BI-WT17	*Phaethon_lepturus*	Bird	2012	ground_nester	1
BI-WT27	*Phaethon_lepturus*	Bird	2012	ground_nester	0
BI-WT28	*Phaethon_lepturus*	Bird	2012	ground_nester	0
BI-WT29	*Phaethon_lepturus*	Bird	2012	ground_nester	0
BI-WT32	*Phaethon_lepturus*	Bird	2012	ground_nester	0
BI-WT33	*Phaethon_lepturus*	Bird	2012	ground_nester	0
BI-WT34	*Phaethon_lepturus*	Bird	2012	ground_nester	0
BI-WT35	*Phaethon_lepturus*	Bird	2012	ground_nester	1
15_328	*Anous_stolidus*	Cousin	2015	ground_nester	0
15_329	*Anous_stolidus*	Cousin	2015	ground_nester	1
15_330	*Anous_stolidus*	Cousin	2015	ground_nester	1
15_331	*Anous_stolidus*	Cousin	2015	ground_nester	0
15_332	*Anous_stolidus*	Cousin	2015	ground_nester	0

15_333	*Anous_stolidus*	Cousin	2015	ground_nester	1
15_335	*Anous_stolidus*	Cousin	2015	ground_nester	0
15_336	*Anous_stolidus*	Cousin	2015	ground_nester	1
15_337	*Anous_stolidus*	Cousin	2015	ground_nester	0
15_338	*Anous_stolidus*	Cousin	2015	ground_nester	0
15_339	*Anous_stolidus*	Cousin	2015	ground_nester	0
15_352	*Anous_stolidus*	Cousin	2015	ground_nester	1
15_353	*Anous_stolidus*	Cousin	2015	ground_nester	1
15_354	*Anous_stolidus*	Cousin	2015	ground_nester	1
15_355	*Anous_stolidus*	Cousin	2015	ground_nester	0
15_356	*Anous_stolidus*	Cousin	2015	ground_nester	1
15_357	*Anous_stolidus*	Cousin	2015	ground_nester	0
15_358	*Anous_stolidus*	Cousin	2015	ground_nester	0
15_359	*Anous_stolidus*	Cousin	2015	ground_nester	0
15_360	*Anous_stolidus*	Cousin	2015	ground_nester	1
15_361	*Anous_stolidus*	Cousin	2015	ground_nester	1
15_362	*Anous_stolidus*	Cousin	2015	ground_nester	0
15_363	*Anous_stolidus*	Cousin	2015	ground_nester	0
15_387	*Anous_stolidus*	Cousin	2015	ground_nester	0
15_388	*Anous_stolidus*	Cousin	2015	ground_nester	1
15_389	*Anous_stolidus*	Cousin	2015	ground_nester	0
15_390	*Anous_stolidus*	Cousin	2015	ground_nester	0
15_391	*Anous_stolidus*	Cousin	2015	ground_nester	1
15_392	*Anous_stolidus*	Cousin	2015	ground_nester	1
15_341	*Anous_tenuirostris*	Cousin	2015	tree-nesters	0
15_342	*Anous_tenuirostris*	Cousin	2015	tree-nesters	0
15_343	*Anous_tenuirostris*	Cousin	2015	tree-nesters	0
15_344	*Anous_tenuirostris*	Cousin	2015	tree-nesters	0
15_345	*Anous_tenuirostris*	Cousin	2015	tree-nesters	1
15_347	*Anous_tenuirostris*	Cousin	2015	tree-nesters	0
15_348	*Anous_tenuirostris*	Cousin	2015	tree-nesters	0
15_349	*Anous_tenuirostris*	Cousin	2015	tree-nesters	0
15_350	*Anous_tenuirostris*	Cousin	2015	tree-nesters	0
15_351	*Anous_tenuirostris*	Cousin	2015	tree-nesters	1
15_364	*Anous_tenuirostris*	Cousin	2015	tree-nesters	0
15_393	*Anous_tenuirostris*	Cousin	2015	tree-nesters	0
15_394	*Anous_tenuirostris*	Cousin	2015	tree-nesters	0
15_396	*Anous_tenuirostris*	Cousin	2015	tree-nesters	0
15_397	*Anous_tenuirostris*	Cousin	2015	tree-nesters	0

15_398	*Anous_tenuirostris*	Cousin	2015	tree-nesters	0
15_399	*Anous_tenuirostris*	Cousin	2015	tree-nesters	1
15_400	*Anous_tenuirostris*	Cousin	2015	tree-nesters	0
15_401	*Anous_tenuirostris*	Cousin	2015	tree-nesters	0
15_405	*Anous_tenuirostris*	Cousin	2015	tree-nesters	1
15_406	*Anous_tenuirostris*	Cousin	2015	tree-nesters	0
15_407	*Anous_tenuirostris*	Cousin	2015	tree-nesters	0
CO-WS-002	*Ardenna_pacifica*	Cousin	2011	ground_nester	0
CO-WS-007	*Ardenna_pacifica*	Cousin	2011	ground_nester	0
CO-WS-008	*Ardenna_pacifica*	Cousin	2011	ground_nester	0
CO-WS-009	*Ardenna_pacifica*	Cousin	2011	ground_nester	0
CO-WS-010	*Ardenna_pacifica*	Cousin	2011	ground_nester	0
CO-WS-011	*Ardenna_pacifica*	Cousin	2011	ground_nester	0
CO-WS-012	*Ardenna_pacifica*	Cousin	2011	ground_nester	0
CO-WS-013	*Ardenna_pacifica*	Cousin	2011	ground_nester	0
CO-WS-014	*Ardenna_pacifica*	Cousin	2011	ground_nester	0
CO-WS-015	*Ardenna_pacifica*	Cousin	2011	ground_nester	0
CO-WS-017	*Ardenna_pacifica*	Cousin	2011	ground_nester	0
CO-WS-018	*Ardenna_pacifica*	Cousin	2011	ground_nester	0
CO-WS-019	*Ardenna_pacifica*	Cousin	2011	ground_nester	0
CO-WS-020	*Ardenna_pacifica*	Cousin	2011	ground_nester	0
CO-WS-021	*Ardenna_pacifica*	Cousin	2011	ground_nester	0
CO-WS-022	*Ardenna_pacifica*	Cousin	2011	ground_nester	0
CO-WS-024	*Ardenna_pacifica*	Cousin	2011	ground_nester	0
CO-WS-027	*Ardenna_pacifica*	Cousin	2011	ground_nester	0
CO-WS-028	*Ardenna_pacifica*	Cousin	2011	ground_nester	0
CO-WS-029	*Ardenna_pacifica*	Cousin	2011	ground_nester	0
CO-WS-031	*Ardenna_pacifica*	Cousin	2011	ground_nester	0
CO-WS-032	*Ardenna_pacifica*	Cousin	2011	ground_nester	0
CO-WS-033	*Ardenna_pacifica*	Cousin	2011	ground_nester	0
CO-WS-035	*Ardenna_pacifica*	Cousin	2011	ground_nester	0
CO-WS-037	*Ardenna_pacifica*	Cousin	2011	ground_nester	0
CO-WS-039	*Ardenna_pacifica*	Cousin	2011	ground_nester	0
CO-WS-041	*Ardenna_pacifica*	Cousin	2011	ground_nester	0
CO-WS-043	*Ardenna_pacifica*	Cousin	2011	ground_nester	0
CO-WS-046	*Ardenna_pacifica*	Cousin	2011	ground_nester	1
CO-WS-047	*Ardenna_pacifica*	Cousin	2011	ground_nester	0
CO-WS-050	*Ardenna_pacifica*	Cousin	2011	ground_nester	0
15_368	*Onychoprion_anaethetus*	Cousin	2015	ground_nester	1

15_369	*Onychoprion_anaethetus*	Cousin	2015	ground_nester	0
15_370	*Onychoprion_anaethetus*	Cousin	2015	ground_nester	0
15_371	*Onychoprion_anaethetus*	Cousin	2015	ground_nester	0
15_372	*Onychoprion_anaethetus*	Cousin	2015	ground_nester	1
15_373	*Onychoprion_anaethetus*	Cousin	2015	ground_nester	0
15_374	*Onychoprion_anaethetus*	Cousin	2015	ground_nester	1
15_375	*Onychoprion_anaethetus*	Cousin	2015	ground_nester	0
15_376	*Onychoprion_anaethetus*	Cousin	2015	ground_nester	1
15_377	*Onychoprion_anaethetus*	Cousin	2015	ground_nester	1
15_378	*Onychoprion_anaethetus*	Cousin	2015	ground_nester	1
15_379	*Onychoprion_anaethetus*	Cousin	2015	ground_nester	1
15_380	*Onychoprion_anaethetus*	Cousin	2015	ground_nester	0
15_381	*Onychoprion_anaethetus*	Cousin	2015	ground_nester	0
15_382	*Onychoprion_anaethetus*	Cousin	2015	ground_nester	0
15_383	*Onychoprion_anaethetus*	Cousin	2015	ground_nester	1
15_386	*Onychoprion_anaethetus*	Cousin	2015	ground_nester	1
CO-WT-002	*Phaethon_lepturus*	Cousin	2011	ground_nester	0
CO-WT-003	*Phaethon_lepturus*	Cousin	2011	ground_nester	0
CO-WT-004	*Phaethon_lepturus*	Cousin	2011	ground_nester	0
CO-WT-006	*Phaethon_lepturus*	Cousin	2011	ground_nester	0
CO-WT-007	*Phaethon_lepturus*	Cousin	2011	ground_nester	0
CO-WT-008	*Phaethon_lepturus*	Cousin	2011	ground_nester	0
CO-WT-011	*Phaethon_lepturus*	Cousin	2011	ground_nester	0
CO-WT-012	*Phaethon_lepturus*	Cousin	2011	ground_nester	0
CO-WT-017	*Phaethon_lepturus*	Cousin	2011	ground_nester	0
CO-WT-020	*Phaethon_lepturus*	Cousin	2011	ground_nester	0
CO-WT-021	*Phaethon_lepturus*	Cousin	2011	ground_nester	0
CO-WT-027	*Phaethon_lepturus*	Cousin	2011	ground_nester	0
CO-WT-034	*Phaethon_lepturus*	Cousin	2011	ground_nester	0
CO-WT-035	*Phaethon_lepturus*	Cousin	2011	ground_nester	0
CO-WT-037	*Phaethon_lepturus*	Cousin	2011	ground_nester	0
CO-WT-039	*Phaethon_lepturus*	Cousin	2011	ground_nester	0
CO-WT-048	*Phaethon_lepturus*	Cousin	2011	ground_nester	0
CO-WT-050	*Phaethon_lepturus*	Cousin	2011	ground_nester	0
15_408	*Anous_stolidus*	Cousine	2015	ground_nester	1
15_409	*Anous_stolidus*	Cousine	2015	ground_nester	0
15_410	*Anous_stolidus*	Cousine	2015	ground_nester	0
15_411	*Anous_stolidus*	Cousine	2015	ground_nester	1
15_416	*Anous_stolidus*	Cousine	2015	ground_nester	1

15_417	*Anous_stolidus*	Cousine	2015	ground_nester	1
15_419	*Anous_stolidus*	Cousine	2015	ground_nester	0
15_420	*Anous_stolidus*	Cousine	2015	ground_nester	0
15_421	*Anous_stolidus*	Cousine	2015	ground_nester	0
15_422	*Anous_stolidus*	Cousine	2015	ground_nester	1
15_423	*Anous_stolidus*	Cousine	2015	ground_nester	0
15_424	*Anous_stolidus*	Cousine	2015	ground_nester	0
15_446	*Anous_stolidus*	Cousine	2015	ground_nester	0
15_483	*Anous_stolidus*	Cousine	2015	ground_nester	0
15_484	*Anous_stolidus*	Cousine	2015	ground_nester	1
15_485	*Anous_stolidus*	Cousine	2015	ground_nester	0
15_486	*Anous_stolidus*	Cousine	2015	ground_nester	1
15_487	*Anous_stolidus*	Cousine	2015	ground_nester	1
15_489	*Anous_stolidus*	Cousine	2015	ground_nester	0
15_490	*Anous_stolidus*	Cousine	2015	ground_nester	0
15_491	*Anous_stolidus*	Cousine	2015	ground_nester	0
15_492	*Anous_stolidus*	Cousine	2015	ground_nester	0
15_493	*Anous_stolidus*	Cousine	2015	ground_nester	0
15_494	*Anous_stolidus*	Cousine	2015	ground_nester	0
15_499	*Anous_stolidus*	Cousine	2015	ground_nester	0
15_500	*Anous_stolidus*	Cousine	2015	ground_nester	0
15_501	*Anous_stolidus*	Cousine	2015	ground_nester	0
15_502	*Anous_stolidus*	Cousine	2015	ground_nester	0
15_412	*Anous_tenuirostris*	Cousine	2015	tree-nesters	0
15_413	*Anous_tenuirostris*	Cousine	2015	tree-nesters	0
15_425	*Anous_tenuirostris*	Cousine	2015	tree-nesters	0
15_428	*Anous_tenuirostris*	Cousine	2015	tree-nesters	0
15_430	*Anous_tenuirostris*	Cousine	2015	tree-nesters	0
15_431	*Anous_tenuirostris*	Cousine	2015	tree-nesters	0
15_432	*Anous_tenuirostris*	Cousine	2015	tree-nesters	0
15_433	*Anous_tenuirostris*	Cousine	2015	tree-nesters	0
15_434	*Anous_tenuirostris*	Cousine	2015	tree-nesters	0
15_435	*Anous_tenuirostris*	Cousine	2015	tree-nesters	0
15_437	*Anous_tenuirostris*	Cousine	2015	tree-nesters	0
15_438	*Anous_tenuirostris*	Cousine	2015	tree-nesters	0
15_439	*Anous_tenuirostris*	Cousine	2015	tree-nesters	0
15_440	*Anous_tenuirostris*	Cousine	2015	tree-nesters	0
15_441	*Anous_tenuirostris*	Cousine	2015	tree-nesters	0
15_442	*Anous_tenuirostris*	Cousine	2015	tree-nesters	0

15_444	*Anous_tenuirostris*	Cousine	2015	tree-nesters	0
15_445	*Anous_tenuirostris*	Cousine	2015	tree-nesters	0
15_461	*Anous_tenuirostris*	Cousine	2015	tree-nesters	0
15_468	*Anous_tenuirostris*	Cousine	2015	tree-nesters	1
15_471	*Anous_tenuirostris*	Cousine	2015	tree-nesters	0
15_472	*Anous_tenuirostris*	Cousine	2015	tree-nesters	0
15_473	*Anous_tenuirostris*	Cousine	2015	tree-nesters	0
15_480	*Anous_tenuirostris*	Cousine	2015	tree-nesters	0
15_482	*Anous_tenuirostris*	Cousine	2015	tree-nesters	0
15_504	*Anous_tenuirostris*	Cousine	2015	tree-nesters	0
15_505	*Anous_tenuirostris*	Cousine	2015	tree-nesters	0
15_506	*Anous_tenuirostris*	Cousine	2015	tree-nesters	0
15_507	*Anous_tenuirostris*	Cousine	2015	tree-nesters	0
15_508	*Anous_tenuirostris*	Cousine	2015	tree-nesters	0
15_509	*Anous_tenuirostris*	Cousine	2015	tree-nesters	0
15_447	*Ardenna_pacifica*	Cousine	2015	ground_nester	0
15_449	*Ardenna_pacifica*	Cousine	2015	ground_nester	0
15_450	*Ardenna_pacifica*	Cousine	2015	ground_nester	0
15_451	*Ardenna_pacifica*	Cousine	2015	ground_nester	0
15_453	*Ardenna_pacifica*	Cousine	2015	ground_nester	0
15_454	*Ardenna_pacifica*	Cousine	2015	ground_nester	0
15_455	*Ardenna_pacifica*	Cousine	2015	ground_nester	0
15_458	*Ardenna_pacifica*	Cousine	2015	ground_nester	0
15_464	*Ardenna_pacifica*	Cousine	2015	ground_nester	0
15_465	*Ardenna_pacifica*	Cousine	2015	ground_nester	0
15_474	*Ardenna_pacifica*	Cousine	2015	ground_nester	0
15_475	*Ardenna_pacifica*	Cousine	2015	ground_nester	0
15_495	*Ardenna_pacifica*	Cousine	2015	ground_nester	0
15_496	*Ardenna_pacifica*	Cousine	2015	ground_nester	0
15_497	*Ardenna_pacifica*	Cousine	2015	ground_nester	0
15_498	*Ardenna_pacifica*	Cousine	2015	ground_nester	0
15_510	*Ardenna_pacifica*	Cousine	2015	ground_nester	0
15_511	*Ardenna_pacifica*	Cousine	2015	ground_nester	0
15_513	*Ardenna_pacifica*	Cousine	2015	ground_nester	0
15_514	*Ardenna_pacifica*	Cousine	2015	ground_nester	0
15_515	*Ardenna_pacifica*	Cousine	2015	ground_nester	0
15_517	*Ardenna_pacifica*	Cousine	2015	ground_nester	0
15_518	*Ardenna_pacifica*	Cousine	2015	ground_nester	0
15_519	*Ardenna_pacifica*	Cousine	2015	ground_nester	0

EU-GF-019	*Fregata_minor*	Europa	2012	tree-nesters	0
EU-GF-021	*Fregata_minor*	Europa	2012	tree-nesters	0
EU-GF-024	*Fregata_minor*	Europa	2012	tree-nesters	0
EU-GF-025	*Fregata_minor*	Europa	2012	tree-nesters	0
EU-GF-033	*Fregata_minor*	Europa	2012	tree-nesters	0
EU-GF-034	*Fregata_minor*	Europa	2012	tree-nesters	0
EU-GF-036	*Fregata_minor*	Europa	2012	tree-nesters	0
EU-GF-041	*Fregata_minor*	Europa	2012	tree-nesters	0
EU-GF-042	*Fregata_minor*	Europa	2012	tree-nesters	0
EU-GF-043	*Fregata_minor*	Europa	2012	tree-nesters	0
EU-GF-045	*Fregata_minor*	Europa	2012	tree-nesters	0
EU-GF-046	*Fregata_minor*	Europa	2012	tree-nesters	0
EU-GF-047	*Fregata_minor*	Europa	2012	tree-nesters	0
EU-GF-048	*Fregata_minor*	Europa	2012	tree-nesters	0
EU-ST-001	*Onychoprion_fuscatus*	Europa	2011	ground_nester	1
EU-ST-002	*Onychoprion_fuscatus*	Europa	2011	ground_nester	0
EU-ST-003	*Onychoprion_fuscatus*	Europa	2011	ground_nester	0
EU-ST-005	*Onychoprion_fuscatus*	Europa	2011	ground_nester	1
EU-ST-006	*Onychoprion_fuscatus*	Europa	2011	ground_nester	0
EU-ST-010	*Onychoprion_fuscatus*	Europa	2011	ground_nester	0
EU-ST-012	*Onychoprion_fuscatus*	Europa	2011	ground_nester	0
EU-ST-013	*Onychoprion_fuscatus*	Europa	2011	ground_nester	0
EU-ST-016	*Onychoprion_fuscatus*	Europa	2011	ground_nester	0
EU-ST-017	*Onychoprion_fuscatus*	Europa	2011	ground_nester	0
EU-ST-018	*Onychoprion_fuscatus*	Europa	2011	ground_nester	0
EU-ST-019	*Onychoprion_fuscatus*	Europa	2011	ground_nester	1
EU-ST-020	*Onychoprion_fuscatus*	Europa	2011	ground_nester	0
EU-ST-023	*Onychoprion_fuscatus*	Europa	2011	ground_nester	0
EU-ST-025	*Onychoprion_fuscatus*	Europa	2011	ground_nester	1
EU-ST-026	*Onychoprion_fuscatus*	Europa	2011	ground_nester	0
EU-ST-027	*Onychoprion_fuscatus*	Europa	2011	ground_nester	0
EU-ST-029	*Onychoprion_fuscatus*	Europa	2011	ground_nester	0
EU-ST-032	*Onychoprion_fuscatus*	Europa	2011	ground_nester	0
EU-ST-037	*Onychoprion_fuscatus*	Europa	2011	ground_nester	0
EU-ST-038	*Onychoprion_fuscatus*	Europa	2011	ground_nester	0
EU-ST-040	*Onychoprion_fuscatus*	Europa	2011	ground_nester	1
EU-ST-042	*Onychoprion_fuscatus*	Europa	2011	ground_nester	0
EU-ST-043	*Onychoprion_fuscatus*	Europa	2011	ground_nester	0
EU-ST-044	*Onychoprion_fuscatus*	Europa	2011	ground_nester	0

EU-ST-045	Onychoprion_fuscatus	Europa	2011	ground_nester	0
EU-ST-046	Onychoprion_fuscatus	Europa	2011	ground_nester	0
EU-ST-047	Onychoprion_fuscatus	Europa	2011	ground_nester	0
EU-ST-049	Onychoprion_fuscatus	Europa	2011	ground_nester	0
EU-ST-050	Onychoprion_fuscatus	Europa	2011	ground_nester	1
EU-ST-051	Onychoprion_fuscatus	Europa	2011	ground_nester	1
EU-ST-052	Onychoprion_fuscatus	Europa	2011	ground_nester	1
EU-ST-053	Onychoprion_fuscatus	Europa	2011	ground_nester	0
EU-ST-054	Onychoprion_fuscatus	Europa	2011	ground_nester	0
EU-ST-055	Onychoprion_fuscatus	Europa	2011	ground_nester	1
EU-ST-057	Onychoprion_fuscatus	Europa	2011	ground_nester	0
EU-ST-058	Onychoprion_fuscatus	Europa	2011	ground_nester	1
EU-ST-059	Onychoprion_fuscatus	Europa	2011	ground_nester	0
EU-ST-060	Onychoprion_fuscatus	Europa	2011	ground_nester	0
EU-ST-061	Onychoprion_fuscatus	Europa	2011	ground_nester	1
EU-ST-065	Onychoprion_fuscatus	Europa	2011	ground_nester	0
EU-ST-066	Onychoprion_fuscatus	Europa	2011	ground_nester	1
EU-ST-069	Onychoprion_fuscatus	Europa	2011	ground_nester	1
EU-ST-070	Onychoprion_fuscatus	Europa	2011	ground_nester	1
EU-ST-074	Onychoprion_fuscatus	Europa	2011	ground_nester	0
EU-ST-076	Onychoprion_fuscatus	Europa	2011	ground_nester	0
EU-ST-077	Onychoprion_fuscatus	Europa	2011	ground_nester	1
EU-ST-078	Onychoprion_fuscatus	Europa	2011	ground_nester	1
EU-ST-079	Onychoprion_fuscatus	Europa	2011	ground_nester	0
EU-ST-080	Onychoprion_fuscatus	Europa	2011	ground_nester	0
EU-ST-081	Onychoprion_fuscatus	Europa	2011	ground_nester	1
EU-ST-082	Onychoprion_fuscatus	Europa	2011	ground_nester	1
EU-ST-083	Onychoprion_fuscatus	Europa	2011	ground_nester	0
EU-ST-086	Onychoprion_fuscatus	Europa	2011	ground_nester	1
EU-ST-087	Onychoprion_fuscatus	Europa	2011	ground_nester	0
EU-ST-090	Onychoprion_fuscatus	Europa	2011	ground_nester	0
EU-ST-091	Onychoprion_fuscatus	Europa	2011	ground_nester	1
EU-ST-092	Onychoprion_fuscatus	Europa	2011	ground_nester	0
EU-ST-093	Onychoprion_fuscatus	Europa	2011	ground_nester	1
EU-ST-094	Onychoprion_fuscatus	Europa	2011	ground_nester	0
EU-ST-098	Onychoprion_fuscatus	Europa	2011	ground_nester	1
EU-ST-099	Onychoprion_fuscatus	Europa	2011	ground_nester	1
EU-ST-100	Onychoprion_fuscatus	Europa	2011	ground_nester	1
EU-ST-102	Onychoprion_fuscatus	Europa	2011	ground_nester	0

EU-ST-103	*Onychoprion_fuscatus*	Europa	2011	ground_nester	0
EU-ST-104	*Onychoprion_fuscatus*	Europa	2011	ground_nester	0
EU-ST-105	*Onychoprion_fuscatus*	Europa	2011	ground_nester	0
EU-ST-106	*Onychoprion_fuscatus*	Europa	2011	ground_nester	1
EU-ST-107	*Onychoprion_fuscatus*	Europa	2011	ground_nester	0
EU-ST-109	*Onychoprion_fuscatus*	Europa	2011	ground_nester	0
EU-ST-110	*Onychoprion_fuscatus*	Europa	2011	ground_nester	0
EU-ST-114	*Onychoprion_fuscatus*	Europa	2011	ground_nester	1
EU-ST-115	*Onychoprion_fuscatus*	Europa	2011	ground_nester	1
EU-ST-116	*Onychoprion_fuscatus*	Europa	2011	ground_nester	0
EU-ST-118	*Onychoprion_fuscatus*	Europa	2011	ground_nester	0
EU-ST-120	*Onychoprion_fuscatus*	Europa	2011	ground_nester	0
EU-ST-124	*Onychoprion_fuscatus*	Europa	2011	ground_nester	1
EU-ST-125	*Onychoprion_fuscatus*	Europa	2011	ground_nester	0
EU-ST-126	*Onychoprion_fuscatus*	Europa	2011	ground_nester	0
EU-ST-130	*Onychoprion_fuscatus*	Europa	2011	ground_nester	0
EU-ST-131	*Onychoprion_fuscatus*	Europa	2011	ground_nester	0
EU-ST-132	*Onychoprion_fuscatus*	Europa	2011	ground_nester	1
EU-ST-133	*Onychoprion_fuscatus*	Europa	2011	ground_nester	0
EU-ST-134	*Onychoprion_fuscatus*	Europa	2011	ground_nester	1
EU-ST-135	*Onychoprion_fuscatus*	Europa	2011	ground_nester	0
EU-ST-136	*Onychoprion_fuscatus*	Europa	2011	ground_nester	0
EU-ST-139	*Onychoprion_fuscatus*	Europa	2011	ground_nester	0
EU-ST-140	*Onychoprion_fuscatus*	Europa	2011	ground_nester	1
EU-ST-141	*Onychoprion_fuscatus*	Europa	2011	ground_nester	0
EU-ST-143	*Onychoprion_fuscatus*	Europa	2011	ground_nester	0
EU-ST-145	*Onychoprion_fuscatus*	Europa	2011	ground_nester	0
EU-ST-147	*Onychoprion_fuscatus*	Europa	2011	ground_nester	1
EU-ST-148	*Onychoprion_fuscatus*	Europa	2011	ground_nester	0
EU-ST-149	*Onychoprion_fuscatus*	Europa	2011	ground_nester	0
EU-ST-150	*Onychoprion_fuscatus*	Europa	2011	ground_nester	1
EU-ST-152	*Onychoprion_fuscatus*	Europa	2011	ground_nester	0
EU-ST-154	*Onychoprion_fuscatus*	Europa	2011	ground_nester	0
EU-ST-156	*Onychoprion_fuscatus*	Europa	2011	ground_nester	1
EU-ST-157	*Onychoprion_fuscatus*	Europa	2011	ground_nester	0
EU-ST-161	*Onychoprion_fuscatus*	Europa	2011	ground_nester	0
EU-ST-163	*Onychoprion_fuscatus*	Europa	2011	ground_nester	0
EU-ST-164	*Onychoprion_fuscatus*	Europa	2011	ground_nester	0
EU-ST-165	*Onychoprion_fuscatus*	Europa	2011	ground_nester	1

EU-ST-167	*Onychoprion_fuscatus*	Europa	2011	ground_nester	1
EU-ST-168	*Onychoprion_fuscatus*	Europa	2011	ground_nester	1
EU-ST-170	*Onychoprion_fuscatus*	Europa	2011	ground_nester	1
EU-ST-171	*Onychoprion_fuscatus*	Europa	2011	ground_nester	1
EU-ST-172	*Onychoprion_fuscatus*	Europa	2011	ground_nester	0
EU-ST-173	*Onychoprion_fuscatus*	Europa	2011	ground_nester	0
EU-ST-174	*Onychoprion_fuscatus*	Europa	2011	ground_nester	0
EU-ST-175	*Onychoprion_fuscatus*	Europa	2011	ground_nester	0
EU-ST-176	*Onychoprion_fuscatus*	Europa	2011	ground_nester	0
EU-ST-177	*Onychoprion_fuscatus*	Europa	2011	ground_nester	0
EU-ST-179	*Onychoprion_fuscatus*	Europa	2011	ground_nester	0
EU-ST-180	*Onychoprion_fuscatus*	Europa	2011	ground_nester	0
EU-ST-181	*Onychoprion_fuscatus*	Europa	2011	ground_nester	0
EU-ST-182	*Onychoprion_fuscatus*	Europa	2011	ground_nester	1
EU-ST-183	*Onychoprion_fuscatus*	Europa	2011	ground_nester	1
EU-ST-185	*Onychoprion_fuscatus*	Europa	2011	ground_nester	1
EU-ST-186	*Onychoprion_fuscatus*	Europa	2011	ground_nester	0
EU-ST-187	*Onychoprion_fuscatus*	Europa	2011	ground_nester	1
EU-ST-188	*Onychoprion_fuscatus*	Europa	2011	ground_nester	0
EU-ST-190	*Onychoprion_fuscatus*	Europa	2011	ground_nester	0
EU-ST-191	*Onychoprion_fuscatus*	Europa	2011	ground_nester	0
EU-ST-193	*Onychoprion_fuscatus*	Europa	2011	ground_nester	0
EU-ST-194	*Onychoprion_fuscatus*	Europa	2011	ground_nester	0
EU-ST-195	*Onychoprion_fuscatus*	Europa	2011	ground_nester	0
EU-ST-197	*Onychoprion_fuscatus*	Europa	2011	ground_nester	1
EU-ST-198	*Onychoprion_fuscatus*	Europa	2011	ground_nester	0
EU-ST-199	*Onychoprion_fuscatus*	Europa	2011	ground_nester	0
EU-ST-200	*Onychoprion_fuscatus*	Europa	2011	ground_nester	0
EU-ST-201	*Onychoprion_fuscatus*	Europa	2011	ground_nester	0
EU-ST-202	*Onychoprion_fuscatus*	Europa	2011	ground_nester	0
EU-ST-225	*Onychoprion_fuscatus*	Europa	2012	ground_nester	0
EU-ST-226	*Onychoprion_fuscatus*	Europa	2012	ground_nester	0
EU-ST-231	*Onychoprion_fuscatus*	Europa	2012	ground_nester	0
EU-ST-232	*Onychoprion_fuscatus*	Europa	2012	ground_nester	1
EU-ST-251	*Onychoprion_fuscatus*	Europa	2012	ground_nester	0
EU-WT-002	*Phaethon_lepturus*	Europa	2011	ground_nester	0
EU-WT-003	*Phaethon_lepturus*	Europa	2011	ground_nester	0
EU-WT-004	*Phaethon_lepturus*	Europa	2011	ground_nester	0
EU-WT-005	*Phaethon_lepturus*	Europa	2011	ground_nester	0

EU-WT-007	*Phaethon_lepturus*	Europa	2011	ground_nester	0
EU-WT-008	*Phaethon_lepturus*	Europa	2011	ground_nester	0
EU-WT-010	*Phaethon_lepturus*	Europa	2011	ground_nester	0
EU-WT-011	*Phaethon_lepturus*	Europa	2011	ground_nester	0
EU-WT-016	*Phaethon_lepturus*	Europa	2011	ground_nester	0
EU-WT-017	*Phaethon_lepturus*	Europa	2011	ground_nester	1
EU-WT-018	*Phaethon_lepturus*	Europa	2011	ground_nester	0
EU-WT-019	*Phaethon_lepturus*	Europa	2011	ground_nester	0
EU-WT-020	*Phaethon_lepturus*	Europa	2011	ground_nester	0
EU-WT-022	*Phaethon_lepturus*	Europa	2011	ground_nester	1
EU-WT-024	*Phaethon_lepturus*	Europa	2011	ground_nester	0
EU-WT-025	*Phaethon_lepturus*	Europa	2011	ground_nester	1
EU-WT-026	*Phaethon_lepturus*	Europa	2011	ground_nester	0
EU-WT-027	*Phaethon_lepturus*	Europa	2011	ground_nester	0
EU-WT-028	*Phaethon_lepturus*	Europa	2011	ground_nester	1
EU-WT-029	*Phaethon_lepturus*	Europa	2011	ground_nester	0
EU-WT-030	*Phaethon_lepturus*	Europa	2011	ground_nester	0
EU-WT-031	*Phaethon_lepturus*	Europa	2011	ground_nester	0
EU-WT-035	*Phaethon_lepturus*	Europa	2011	ground_nester	0
EU-WT-037	*Phaethon_lepturus*	Europa	2011	ground_nester	0
EU-WT-038	*Phaethon_lepturus*	Europa	2011	ground_nester	1
EU-WT-039	*Phaethon_lepturus*	Europa	2011	ground_nester	0
EU-WT-040	*Phaethon_lepturus*	Europa	2011	ground_nester	1
EU-WT-041	*Phaethon_lepturus*	Europa	2011	ground_nester	0
EU-WT-042	*Phaethon_lepturus*	Europa	2011	ground_nester	0
EU-WT-043	*Phaethon_lepturus*	Europa	2011	ground_nester	0
EU-WT-044	*Phaethon_lepturus*	Europa	2011	ground_nester	0
EU-RT-003	*Phaethon_rubricauda*	Europa	2011	ground_nester	0
EU-RT-007	*Phaethon_rubricauda*	Europa	2011	ground_nester	0
EU-RT-010	*Phaethon_rubricauda*	Europa	2011	ground_nester	0
EU-RT-011	*Phaethon_rubricauda*	Europa	2011	ground_nester	0
EU-RT-012	*Phaethon_rubricauda*	Europa	2011	ground_nester	0
EU-RT-014	*Phaethon_rubricauda*	Europa	2011	ground_nester	0
EU-RT-015	*Phaethon_rubricauda*	Europa	2011	ground_nester	0
EU-RT-021	*Phaethon_rubricauda*	Europa	2011	ground_nester	0
EU-RT-022	*Phaethon_rubricauda*	Europa	2011	ground_nester	0
EU-RT-023	*Phaethon_rubricauda*	Europa	2011	ground_nester	0
EU-RT-024	*Phaethon_rubricauda*	Europa	2011	ground_nester	0
EU-RT-025	*Phaethon_rubricauda*	Europa	2011	ground_nester	0

EU-RT-026	*Phaethon_rubricauda*	Europa	2011	ground_nester	0
EU-RT-027	*Phaethon_rubricauda*	Europa	2011	ground_nester	0
EU-RT-028	*Phaethon_rubricauda*	Europa	2011	ground_nester	0
EU-RT-029	*Phaethon_rubricauda*	Europa	2011	ground_nester	0
EU-RT-030	*Phaethon_rubricauda*	Europa	2011	ground_nester	0
EU-RT-031	*Phaethon_rubricauda*	Europa	2011	ground_nester	0
EU-RT-032	*Phaethon_rubricauda*	Europa	2011	ground_nester	0
EU-RT-033	*Phaethon_rubricauda*	Europa	2011	ground_nester	1
EU-RT-034	*Phaethon_rubricauda*	Europa	2011	ground_nester	0
EU-RT-035	*Phaethon_rubricauda*	Europa	2011	ground_nester	0
EU-RT-036	*Phaethon_rubricauda*	Europa	2011	ground_nester	0
EU-RT-038	*Phaethon_rubricauda*	Europa	2011	ground_nester	1
EU-RT-039	*Phaethon_rubricauda*	Europa	2011	ground_nester	0
EU-RT-040	*Phaethon_rubricauda*	Europa	2011	ground_nester	0
EU-RT-042	*Phaethon_rubricauda*	Europa	2011	ground_nester	0
EU-RT-043	*Phaethon_rubricauda*	Europa	2011	ground_nester	0
EU-RT-044	*Phaethon_rubricauda*	Europa	2011	ground_nester	0
EU-RT-045	*Phaethon_rubricauda*	Europa	2011	ground_nester	0
EU-RT-046	*Phaethon_rubricauda*	Europa	2011	ground_nester	0
EU-RT-047	*Phaethon_rubricauda*	Europa	2011	ground_nester	0
EU-RT-048	*Phaethon_rubricauda*	Europa	2011	ground_nester	0
EU-RT-049	*Phaethon_rubricauda*	Europa	2011	ground_nester	0
EU-RB-001	*Sula_sula*	Europa	2011	tree-nesters	0
EU-RB-004	*Sula_sula*	Europa	2011	tree-nesters	0
EU-RB-005	*Sula_sula*	Europa	2011	tree-nesters	0
EU-RB-006	*Sula_sula*	Europa	2011	tree-nesters	1
EU-RB-007	*Sula_sula*	Europa	2011	tree-nesters	0
EU-RB-008	*Sula_sula*	Europa	2011	tree-nesters	0
EU-RB-010	*Sula_sula*	Europa	2011	tree-nesters	0
EU-RB-012	*Sula_sula*	Europa	2011	tree-nesters	0
EU-RB-013	*Sula_sula*	Europa	2011	tree-nesters	0
EU-RB-015	*Sula_sula*	Europa	2011	tree-nesters	0
EU-RB-016	*Sula_sula*	Europa	2011	tree-nesters	0
EU-RB-017	*Sula_sula*	Europa	2011	tree-nesters	0
EU-RB-018	*Sula_sula*	Europa	2011	tree-nesters	0
EU-RB-019	*Sula_sula*	Europa	2011	tree-nesters	0
EU-RB-020	*Sula_sula*	Europa	2011	tree-nesters	0
EU-RB-023	*Sula_sula*	Europa	2011	tree-nesters	0
EU-RB-024	*Sula_sula*	Europa	2011	tree-nesters	1

EU-RB-025	*Sula_sula*	Europa	2011	tree-nesters	0
EU-RB-026	*Sula_sula*	Europa	2011	tree-nesters	0
EU-RB-027	*Sula_sula*	Europa	2011	tree-nesters	0
EU-RB-028	*Sula_sula*	Europa	2011	tree-nesters	0
EU-RB-029	*Sula_sula*	Europa	2011	tree-nesters	0
EU-RB-030	*Sula_sula*	Europa	2011	tree-nesters	0
EU-RB-031	*Sula_sula*	Europa	2012	tree-nesters	0
EU-RB-038	*Sula_sula*	Europa	2012	tree-nesters	0
EU-RB-039	*Sula_sula*	Europa	2012	tree-nesters	0
EU-RB-040	*Sula_sula*	Europa	2012	tree-nesters	0
EU-RB-041	*Sula_sula*	Europa	2012	tree-nesters	0
EU-RB-042	*Sula_sula*	Europa	2012	tree-nesters	0
EU-RB-044	*Sula_sula*	Europa	2012	tree-nesters	0
EU-RB-049	*Sula_sula*	Europa	2012	tree-nesters	0
EU-RB-050	*Sula_sula*	Europa	2012	tree-nesters	0
EU-RB-052	*Sula_sula*	Europa	2012	tree-nesters	0
EU-RB-054	*Sula_sula*	Europa	2012	tree-nesters	0
EU-RB-057	*Sula_sula*	Europa	2012	tree-nesters	0
EU-RB-058	*Sula_sula*	Europa	2012	tree-nesters	1
EU-GF-001	*Fregata_minor*	Europa	2011	tree-nesters	0
EU-GF-002	*Fregata_minor*	Europa	2011	tree-nesters	0
EU-GF-003	*Fregata_minor*	Europa	2011	tree-nesters	0
EU-GF-004	*Fregata_minor*	Europa	2011	tree-nesters	0
EU-GF-005	*Fregata_minor*	Europa	2011	tree-nesters	0
EU-GF-006	*Fregata_minor*	Europa	2011	tree-nesters	0
EU-GF-007	*Fregata_minor*	Europa	2011	tree-nesters	0
EU-GF-009	*Fregata_minor*	Europa	2011	tree-nesters	0
EU-GF-010	*Fregata_minor*	Europa	2011	tree-nesters	0
EU-GF-011	*Fregata_minor*	Europa	2011	tree-nesters	0
EU-GF-012	*Fregata_minor*	Europa	2011	tree-nesters	0
EU-GF-013	*Fregata_minor*	Europa	2011	tree-nesters	0
EU-GF-014	*Fregata_minor*	Europa	2011	tree-nesters	0
EU-GF-015	*Fregata_minor*	Europa	2011	tree-nesters	0
EU-GF-016	*Fregata_minor*	Europa	2011	tree-nesters	0
EU-GF-017	*Fregata_minor*	Europa	2011	tree-nesters	0
EU-GF-027	*Fregata_minor*	Europa	2012	tree-nesters	0
EU-GF-028	*Fregata_minor*	Europa	2012	tree-nesters	0
EU-GF-029	*Fregata_minor*	Europa	2012	tree-nesters	0
EU-GF-037	*Fregata_minor*	Europa	2012	tree-nesters	0

EU-GF-038	*Fregata_minor*	Europa	2012	tree-nesters	0
EU-GF-040	*Fregata_minor*	Europa	2012	tree-nesters	0
EU-ST-221	*Onychoprion_fuscatus*	Europa	2012	ground_nester	0
EU-ST-223	*Onychoprion_fuscatus*	Europa	2012	ground_nester	0
EU-ST-224	*Onychoprion_fuscatus*	Europa	2012	ground_nester	0
EU-ST-227	*Onychoprion_fuscatus*	Europa	2012	ground_nester	0
EU-ST-228	*Onychoprion_fuscatus*	Europa	2012	ground_nester	0
EU-ST-229	*Onychoprion_fuscatus*	Europa	2012	ground_nester	0
EU-ST-230	*Onychoprion_fuscatus*	Europa	2012	ground_nester	0
EU-ST-235	*Onychoprion_fuscatus*	Europa	2012	ground_nester	0
EU-ST-236	*Onychoprion_fuscatus*	Europa	2012	ground_nester	0
EU-ST-237	*Onychoprion_fuscatus*	Europa	2012	ground_nester	0
EU-ST-238	*Onychoprion_fuscatus*	Europa	2012	ground_nester	0
EU-ST-239	*Onychoprion_fuscatus*	Europa	2012	ground_nester	0
EU-ST-240	*Onychoprion_fuscatus*	Europa	2012	ground_nester	0
EU-ST-242	*Onychoprion_fuscatus*	Europa	2012	ground_nester	0
EU-ST-243	*Onychoprion_fuscatus*	Europa	2012	ground_nester	0
EU-ST-244	*Onychoprion_fuscatus*	Europa	2012	ground_nester	0
EU-ST-245	*Onychoprion_fuscatus*	Europa	2012	ground_nester	0
EU-ST-246	*Onychoprion_fuscatus*	Europa	2012	ground_nester	0
EU-ST-247	*Onychoprion_fuscatus*	Europa	2012	ground_nester	0
EU-ST-248	*Onychoprion_fuscatus*	Europa	2012	ground_nester	0
EU-ST-249	*Onychoprion_fuscatus*	Europa	2012	ground_nester	0
EU-ST-250	*Onychoprion_fuscatus*	Europa	2012	ground_nester	0
EU-ST-252	*Onychoprion_fuscatus*	Europa	2012	ground_nester	0
EU-ST-253	*Onychoprion_fuscatus*	Europa	2012	ground_nester	0
EU-ST-255	*Onychoprion_fuscatus*	Europa	2012	ground_nester	0
EU-ST-256	*Onychoprion_fuscatus*	Europa	2012	ground_nester	0
EU-ST-257	*Onychoprion_fuscatus*	Europa	2012	ground_nester	0
EU-ST-258	*Onychoprion_fuscatus*	Europa	2012	ground_nester	0
EU-ST-259	*Onychoprion_fuscatus*	Europa	2012	ground_nester	0
EU-ST-261	*Onychoprion_fuscatus*	Europa	2012	ground_nester	0
EU-RB-011	*Sula_sula*	Europa	2011	tree-nesters	0
EU-RB-051	*Sula_sula*	Europa	2012	tree-nesters	0
EU-RB-053	*Sula_sula*	Europa	2012	tree-nesters	0
EU-RB-055	*Sula_sula*	Europa	2012	tree-nesters	0
EU-RB-059	*Sula_sula*	Europa	2012	tree-nesters	0
EU-RB-060	*Sula_sula*	Europa	2012	tree-nesters	1
EU-RB-062	*Sula_sula*	Europa	2012	tree-nesters	0

EU-RB-063	*Sula_sula*	Europa	2012	tree-nesters	0
EU-RB-065	*Sula_sula*	Europa	2012	tree-nesters	0
EU-RB-071	*Sula_sula*	Europa	2012	tree-nesters	0
EU-RB-073	*Sula_sula*	Europa	2012	tree-nesters	0
EU-RB-074	*Sula_sula*	Europa	2012	tree-nesters	1
EU-RB-075	*Sula_sula*	Europa	2012	tree-nesters	0
EU-RB-076	*Sula_sula*	Europa	2012	tree-nesters	0
EU-RB-077	*Sula_sula*	Europa	2012	tree-nesters	0
EU-RB-078	*Sula_sula*	Europa	2012	tree-nesters	0
EU-RB-080	*Sula_sula*	Europa	2012	tree-nesters	0
JU-ST-001	*Onychoprion_fuscatus*	Juan_de_Nova	2012	ground_nester	1
JU-ST-004	*Onychoprion_fuscatus*	Juan_de_Nova	2012	ground_nester	0
JU-ST-005	*Onychoprion_fuscatus*	Juan_de_Nova	2012	ground_nester	0
JU-ST-006	*Onychoprion_fuscatus*	Juan_de_Nova	2012	ground_nester	0
JU-ST-007	*Onychoprion_fuscatus*	Juan_de_Nova	2012	ground_nester	0
JU-ST-008	*Onychoprion_fuscatus*	Juan_de_Nova	2012	ground_nester	0
JU-ST-009	*Onychoprion_fuscatus*	Juan_de_Nova	2012	ground_nester	0
JU-ST-011	*Onychoprion_fuscatus*	Juan_de_Nova	2012	ground_nester	0
JU-ST-013	*Onychoprion_fuscatus*	Juan_de_Nova	2012	ground_nester	0
JU-ST-014	*Onychoprion_fuscatus*	Juan_de_Nova	2012	ground_nester	0
JU-ST-016	*Onychoprion_fuscatus*	Juan_de_Nova	2012	ground_nester	0
JU-ST-018	*Onychoprion_fuscatus*	Juan_de_Nova	2012	ground_nester	0
JU-ST-019	*Onychoprion_fuscatus*	Juan_de_Nova	2012	ground_nester	1
JU-ST-020	*Onychoprion_fuscatus*	Juan_de_Nova	2012	ground_nester	0
JU-ST-021	*Onychoprion_fuscatus*	Juan_de_Nova	2012	ground_nester	1
JU-ST-022	*Onychoprion_fuscatus*	Juan_de_Nova	2012	ground_nester	1
JU-ST-025	*Onychoprion_fuscatus*	Juan_de_Nova	2012	ground_nester	0
JU-ST-027	*Onychoprion_fuscatus*	Juan_de_Nova	2012	ground_nester	0
JU-ST-028	*Onychoprion_fuscatus*	Juan_de_Nova	2012	ground_nester	1
JU-ST-030	*Onychoprion_fuscatus*	Juan_de_Nova	2012	ground_nester	0
JU-ST-031	*Onychoprion_fuscatus*	Juan_de_Nova	2012	ground_nester	0
JU-ST-032	*Onychoprion_fuscatus*	Juan_de_Nova	2012	ground_nester	0
JU-ST-034	*Onychoprion_fuscatus*	Juan_de_Nova	2012	ground_nester	0
JU-ST-035	*Onychoprion_fuscatus*	Juan_de_Nova	2012	ground_nester	1
JU-ST-036	*Onychoprion_fuscatus*	Juan_de_Nova	2012	ground_nester	1
JU-ST-037	*Onychoprion_fuscatus*	Juan_de_Nova	2012	ground_nester	0
JU-ST-040	*Onychoprion_fuscatus*	Juan_de_Nova	2012	ground_nester	0
JU-ST-041	*Onychoprion_fuscatus*	Juan_de_Nova	2012	ground_nester	0
JU-ST-042	*Onychoprion_fuscatus*	Juan_de_Nova	2012	ground_nester	0

JU-ST-043	*Onychoprion_fuscatus*	Juan_de_Nova	2012	ground_nester	0
JU-ST-044	*Onychoprion_fuscatus*	Juan_de_Nova	2012	ground_nester	0
JU-ST-047	*Onychoprion_fuscatus*	Juan_de_Nova	2012	ground_nester	1
JU-ST-048	*Onychoprion_fuscatus*	Juan_de_Nova	2012	ground_nester	1
JU-ST-049	*Onychoprion_fuscatus*	Juan_de_Nova	2012	ground_nester	1
JU-ST-051	*Onychoprion_fuscatus*	Juan_de_Nova	2012	ground_nester	0
JU-ST-052	*Onychoprion_fuscatus*	Juan_de_Nova	2012	ground_nester	1
JU-ST-053	*Onychoprion_fuscatus*	Juan_de_Nova	2012	ground_nester	0
JU-ST-055	*Onychoprion_fuscatus*	Juan_de_Nova	2012	ground_nester	0
JU-ST-057	*Onychoprion_fuscatus*	Juan_de_Nova	2012	ground_nester	0
JU-ST-060	*Onychoprion_fuscatus*	Juan_de_Nova	2012	ground_nester	0
JU-ST-061	*Onychoprion_fuscatus*	Juan_de_Nova	2012	ground_nester	0
JU-ST-063	*Onychoprion_fuscatus*	Juan_de_Nova	2012	ground_nester	0
JU-ST-065	*Onychoprion_fuscatus*	Juan_de_Nova	2012	ground_nester	0
JU-ST-066	*Onychoprion_fuscatus*	Juan_de_Nova	2012	ground_nester	0
JU-ST-072	*Onychoprion_fuscatus*	Juan_de_Nova	2012	ground_nester	0
JU-ST-074	*Onychoprion_fuscatus*	Juan_de_Nova	2012	ground_nester	0
JU-ST-075	*Onychoprion_fuscatus*	Juan_de_Nova	2012	ground_nester	0
JU-ST-076	*Onychoprion_fuscatus*	Juan_de_Nova	2012	ground_nester	0
JU-ST-077	*Onychoprion_fuscatus*	Juan_de_Nova	2012	ground_nester	1
JU-ST-085	*Onychoprion_fuscatus*	Juan_de_Nova	2012	ground_nester	0
JU-ST-086	*Onychoprion_fuscatus*	Juan_de_Nova	2012	ground_nester	0
JU-ST-087	*Onychoprion_fuscatus*	Juan_de_Nova	2012	ground_nester	0
JU-ST-089	*Onychoprion_fuscatus*	Juan_de_Nova	2012	ground_nester	0
JU-ST-090	*Onychoprion_fuscatus*	Juan_de_Nova	2012	ground_nester	0
JU-ST-091	*Onychoprion_fuscatus*	Juan_de_Nova	2012	ground_nester	0
JU-ST-092	*Onychoprion_fuscatus*	Juan_de_Nova	2012	ground_nester	0
JU-ST-093	*Onychoprion_fuscatus*	Juan_de_Nova	2012	ground_nester	0
JU-ST-094	*Onychoprion_fuscatus*	Juan_de_Nova	2012	ground_nester	0
JU-ST-095	*Onychoprion_fuscatus*	Juan_de_Nova	2012	ground_nester	0
JU-ST-096	*Onychoprion_fuscatus*	Juan_de_Nova	2012	ground_nester	1
JU-ST-097	*Onychoprion_fuscatus*	Juan_de_Nova	2012	ground_nester	0
JU-ST-098	*Onychoprion_fuscatus*	Juan_de_Nova	2012	ground_nester	0
JU-ST-099	*Onychoprion_fuscatus*	Juan_de_Nova	2012	ground_nester	0
JU-ST-100	*Onychoprion_fuscatus*	Juan_de_Nova	2012	ground_nester	0
JU-ST-201	*Onychoprion_fuscatus*	Juan_de_Nova	2012	ground_nester	0
JU-ST-202	*Onychoprion_fuscatus*	Juan_de_Nova	2012	ground_nester	0
JU-ST-203	*Onychoprion_fuscatus*	Juan_de_Nova	2012	ground_nester	1
JU-ST-204	*Onychoprion_fuscatus*	Juan_de_Nova	2012	ground_nester	0

JU-ST-205	*Onychoprion_fuscatus*	Juan_de_Nova	2012	ground_nester	0
JU-ST-206	*Onychoprion_fuscatus*	Juan_de_Nova	2012	ground_nester	0
JU-ST-207	*Onychoprion_fuscatus*	Juan_de_Nova	2012	ground_nester	0
JU-ST-209	*Onychoprion_fuscatus*	Juan_de_Nova	2012	ground_nester	0
JU-ST-211	*Onychoprion_fuscatus*	Juan_de_Nova	2012	ground_nester	0
JU-ST-212	*Onychoprion_fuscatus*	Juan_de_Nova	2012	ground_nester	0
JU-ST-213	*Onychoprion_fuscatus*	Juan_de_Nova	2012	ground_nester	0
JU-ST-214	*Onychoprion_fuscatus*	Juan_de_Nova	2012	ground_nester	1
JU-ST-215	*Onychoprion_fuscatus*	Juan_de_Nova	2012	ground_nester	0
JU-ST-216	*Onychoprion_fuscatus*	Juan_de_Nova	2012	ground_nester	1
JU-ST-218	*Onychoprion_fuscatus*	Juan_de_Nova	2012	ground_nester	0
JU-ST-219	*Onychoprion_fuscatus*	Juan_de_Nova	2012	ground_nester	0
JU-ST-220	*Onychoprion_fuscatus*	Juan_de_Nova	2012	ground_nester	1
JU-ST-224	*Onychoprion_fuscatus*	Juan_de_Nova	2012	ground_nester	0
JU-ST-225	*Onychoprion_fuscatus*	Juan_de_Nova	2012	ground_nester	0
JU-ST-230	*Onychoprion_fuscatus*	Juan_de_Nova	2012	ground_nester	0
JU-ST-233	*Onychoprion_fuscatus*	Juan_de_Nova	2012	ground_nester	0
JU-ST-235	*Onychoprion_fuscatus*	Juan_de_Nova	2012	ground_nester	0
JU-ST-236	*Onychoprion_fuscatus*	Juan_de_Nova	2012	ground_nester	1
JU-ST-237	*Onychoprion_fuscatus*	Juan_de_Nova	2012	ground_nester	0
JU-ST-242	*Onychoprion_fuscatus*	Juan_de_Nova	2012	ground_nester	0
JU-ST-245	*Onychoprion_fuscatus*	Juan_de_Nova	2012	ground_nester	0
JU-ST-246	*Onychoprion_fuscatus*	Juan_de_Nova	2012	ground_nester	0
JU-ST-248	*Onychoprion_fuscatus*	Juan_de_Nova	2012	ground_nester	0
JU-ST-249	*Onychoprion_fuscatus*	Juan_de_Nova	2012	ground_nester	0
JU-ST-250	*Onychoprion_fuscatus*	Juan_de_Nova	2012	ground_nester	1
JU-ST-253	*Onychoprion_fuscatus*	Juan_de_Nova	2012	ground_nester	0
JU-ST-254	*Onychoprion_fuscatus*	Juan_de_Nova	2012	ground_nester	1
JU-ST-257	*Onychoprion_fuscatus*	Juan_de_Nova	2012	ground_nester	0
JU-ST-260	*Onychoprion_fuscatus*	Juan_de_Nova	2012	ground_nester	0
JU-ST-105	*Onychoprion_fuscatus*	Juan_de_Nova	2012	ground_nester	0
JU-ST-108	*Onychoprion_fuscatus*	Juan_de_Nova	2012	ground_nester	0
JU-ST-110	*Onychoprion_fuscatus*	Juan_de_Nova	2012	ground_nester	0
JU-ST-114	*Onychoprion_fuscatus*	Juan_de_Nova	2012	ground_nester	0
JU-ST-116	*Onychoprion_fuscatus*	Juan_de_Nova	2012	ground_nester	0
JU-ST-117	*Onychoprion_fuscatus*	Juan_de_Nova	2012	ground_nester	0
JU-ST-121	*Onychoprion_fuscatus*	Juan_de_Nova	2012	ground_nester	0
JU-ST-122	*Onychoprion_fuscatus*	Juan_de_Nova	2012	ground_nester	0
JU-ST-124	*Onychoprion_fuscatus*	Juan_de_Nova	2012	ground_nester	0

JU-ST-125	Onychoprion_fuscatus	Juan_de_Nova	2012	ground_nester	0
JU-ST-127	Onychoprion_fuscatus	Juan_de_Nova	2012	ground_nester	0
JU-ST-128	Onychoprion_fuscatus	Juan_de_Nova	2012	ground_nester	0
JU-ST-131	Onychoprion_fuscatus	Juan_de_Nova	2012	ground_nester	0
JU-ST-132	Onychoprion_fuscatus	Juan_de_Nova	2012	ground_nester	0
JU-ST-133	Onychoprion_fuscatus	Juan_de_Nova	2012	ground_nester	0
JU-ST-134	Onychoprion_fuscatus	Juan_de_Nova	2012	ground_nester	0
JU-ST-135	Onychoprion_fuscatus	Juan_de_Nova	2012	ground_nester	0
JU-ST-136	Onychoprion_fuscatus	Juan_de_Nova	2012	ground_nester	0
JU-ST-137	Onychoprion_fuscatus	Juan_de_Nova	2012	ground_nester	0
JU-ST-138	Onychoprion_fuscatus	Juan_de_Nova	2012	ground_nester	0
JU-ST-139	Onychoprion_fuscatus	Juan_de_Nova	2012	ground_nester	0
JU-ST-143	Onychoprion_fuscatus	Juan_de_Nova	2012	ground_nester	0
JU-ST-145	Onychoprion_fuscatus	Juan_de_Nova	2012	ground_nester	0
JU-ST-147	Onychoprion_fuscatus	Juan_de_Nova	2012	ground_nester	0
JU-ST-148	Onychoprion_fuscatus	Juan_de_Nova	2012	ground_nester	0
JU-ST-149	Onychoprion_fuscatus	Juan_de_Nova	2012	ground_nester	0
JU-ST-150	Onychoprion_fuscatus	Juan_de_Nova	2012	ground_nester	0
JU-ST-152	Onychoprion_fuscatus	Juan_de_Nova	2012	ground_nester	0
JU-ST-156	Onychoprion_fuscatus	Juan_de_Nova	2012	ground_nester	0
JU-ST-158	Onychoprion_fuscatus	Juan_de_Nova	2012	ground_nester	0
JU-ST-162	Onychoprion_fuscatus	Juan_de_Nova	2012	ground_nester	0
JU-ST-163	Onychoprion_fuscatus	Juan_de_Nova	2012	ground_nester	0
JU-ST-166	Onychoprion_fuscatus	Juan_de_Nova	2012	ground_nester	0
JU-ST-167	Onychoprion_fuscatus	Juan_de_Nova	2012	ground_nester	0
JU-ST-168	Onychoprion_fuscatus	Juan_de_Nova	2012	ground_nester	0
JU-ST-170	Onychoprion_fuscatus	Juan_de_Nova	2012	ground_nester	0
JU-ST-171	Onychoprion_fuscatus	Juan_de_Nova	2012	ground_nester	0
JU-ST-172	Onychoprion_fuscatus	Juan_de_Nova	2012	ground_nester	0
JU-ST-173	Onychoprion_fuscatus	Juan_de_Nova	2012	ground_nester	0
JU-ST-174	Onychoprion_fuscatus	Juan_de_Nova	2012	ground_nester	0
JU-ST-177	Onychoprion_fuscatus	Juan_de_Nova	2012	ground_nester	0
JU-ST-178	Onychoprion_fuscatus	Juan_de_Nova	2012	ground_nester	0
JU-ST-179	Onychoprion_fuscatus	Juan_de_Nova	2012	ground_nester	0
JU-ST-181	Onychoprion_fuscatus	Juan_de_Nova	2012	ground_nester	0
JU-ST-182	Onychoprion_fuscatus	Juan_de_Nova	2012	ground_nester	0
JU-ST-183	Onychoprion_fuscatus	Juan_de_Nova	2012	ground_nester	0
JU-ST-184	Onychoprion_fuscatus	Juan_de_Nova	2012	ground_nester	0
JU-ST-185	Onychoprion_fuscatus	Juan_de_Nova	2012	ground_nester	0

JU-ST-186	*Onychoprion_fuscatus*	Juan_de_Nova	2012	ground_nester	0
JU-ST-187	*Onychoprion_fuscatus*	Juan_de_Nova	2012	ground_nester	0
JU-ST-188	*Onychoprion_fuscatus*	Juan_de_Nova	2012	ground_nester	0
JU-ST-191	*Onychoprion_fuscatus*	Juan_de_Nova	2012	ground_nester	0
JU-ST-192	*Onychoprion_fuscatus*	Juan_de_Nova	2012	ground_nester	0
JU-ST-193	*Onychoprion_fuscatus*	Juan_de_Nova	2012	ground_nester	0
JU-ST-194	*Onychoprion_fuscatus*	Juan_de_Nova	2012	ground_nester	0
JU-ST-199	*Onychoprion_fuscatus*	Juan_de_Nova	2012	ground_nester	0
JU-ST-200	*Onychoprion_fuscatus*	Juan_de_Nova	2012	ground_nester	0
PIFWS01	*Ardenna_pacifica*	Reunion	2012	ground_nester	0
PIFWS02	*Ardenna_pacifica*	Reunion	2012	ground_nester	0
PIFWS03	*Ardenna_pacifica*	Reunion	2012	ground_nester	0
PIFWS04	*Ardenna_pacifica*	Reunion	2012	ground_nester	0
PIFWS05	*Ardenna_pacifica*	Reunion	2012	ground_nester	1
PIFWS06	*Ardenna_pacifica*	Reunion	2012	ground_nester	0
PIFWS07	*Ardenna_pacifica*	Reunion	2012	ground_nester	0
PIFWS08	*Ardenna_pacifica*	Reunion	2012	ground_nester	0
PIFWS11	*Ardenna_pacifica*	Reunion	2012	ground_nester	0
PIIWS01	*Ardenna_pacifica*	Reunion	2012	ground_nester	0
PIIWS02	*Ardenna_pacifica*	Reunion	2012	ground_nester	0
PIIWS03	*Ardenna_pacifica*	Reunion	2012	ground_nester	0
PIIWS04	*Ardenna_pacifica*	Reunion	2012	ground_nester	0
PIIWS05	*Ardenna_pacifica*	Reunion	2012	ground_nester	0
PIIWS06	*Ardenna_pacifica*	Reunion	2012	ground_nester	0
PIIWS07	*Ardenna_pacifica*	Reunion	2012	ground_nester	0
PIIWS08	*Ardenna_pacifica*	Reunion	2012	ground_nester	0
PIIWS09	*Ardenna_pacifica*	Reunion	2012	ground_nester	0
PIIWS10	*Ardenna_pacifica*	Reunion	2012	ground_nester	1
PIIWS11	*Ardenna_pacifica*	Reunion	2012	ground_nester	0
PIIWS12	*Ardenna_pacifica*	Reunion	2012	ground_nester	0
PIIWS13	*Ardenna_pacifica*	Reunion	2012	ground_nester	0
PIIWS14	*Ardenna_pacifica*	Reunion	2012	ground_nester	1
PIIWS16	*Ardenna_pacifica*	Reunion	2012	ground_nester	1
PIIWS17	*Ardenna_pacifica*	Reunion	2012	ground_nester	0
PIIWS19	*Ardenna_pacifica*	Reunion	2012	ground_nester	0
PIIWS20	*Ardenna_pacifica*	Reunion	2012	ground_nester	0

PIIWS21	Ardenna_pacifica	Reunion	2012	ground_nester	0
PIIWS22	Ardenna_pacifica	Reunion	2012	ground_nester	0
PIIWS23	Ardenna_pacifica	Reunion	2012	ground_nester	0
PIIWS24	Ardenna_pacifica	Reunion	2012	ground_nester	0
PIIWS25	Ardenna_pacifica	Reunion	2012	ground_nester	0
PIIWS26	Ardenna_pacifica	Reunion	2012	ground_nester	0
RUWS01	Ardenna_pacifica	Reunion	2011	ground_nester	0
RUWS02	Ardenna_pacifica	Reunion	2011	ground_nester	0
RUWS04	Ardenna_pacifica	Reunion	2011	ground_nester	0
RUWS05	Ardenna_pacifica	Reunion	2011	ground_nester	0
RUWS06	Ardenna_pacifica	Reunion	2011	ground_nester	0
RUWS07	Ardenna_pacifica	Reunion	2011	ground_nester	0
RUWS08	Ardenna_pacifica	Reunion	2011	ground_nester	0
RUWS12	Ardenna_pacifica	Reunion	2011	ground_nester	0
RUWS15	Ardenna_pacifica	Reunion	2011	ground_nester	0
RUWS16	Ardenna_pacifica	Reunion	2011	ground_nester	0
RUWS17	Ardenna_pacifica	Reunion	2011	ground_nester	0
RUWS18	Ardenna_pacifica	Reunion	2011	ground_nester	1
RUWS19	Ardenna_pacifica	Reunion	2011	ground_nester	0
RUWS20	Ardenna_pacifica	Reunion	2011	ground_nester	0
RUWS21	Ardenna_pacifica	Reunion	2011	ground_nester	0
RUWS22	Ardenna_pacifica	Reunion	2011	ground_nester	0
RUWS23	Ardenna_pacifica	Reunion	2011	ground_nester	0
53_avant_droit	Ardenna_pacifica	Reunion	2013	ground_nester	1
53_avant_gauche	Ardenna_pacifica	Reunion	2013	ground_nester	0
53_fond_droit	Ardenna_pacifica	Reunion	2013	ground_nester	0
53_fond_gauche	Ardenna_pacifica	Reunion	2013	ground_nester	0
PIFWS20	Ardenna_pacifica	Reunion	2013	ground_nester	0
PIFWS21	Ardenna_pacifica	Reunion	2013	ground_nester	0
PIFWS22	Ardenna_pacifica	Reunion	2013	ground_nester	0
PIFWS23	Ardenna_pacifica	Reunion	2013	ground_nester	0
PIFWS24	Ardenna_pacifica	Reunion	2013	ground_nester	0
PIFWS25	Ardenna_pacifica	Reunion	2013	ground_nester	0
PIFWS26	Ardenna_pacifica	Reunion	2013	ground_nester	0
PIFWS27	Ardenna_pacifica	Reunion	2013	ground_nester	0
PIFWS28	Ardenna_pacifica	Reunion	2013	ground_nester	0
PIFWS29	Ardenna_pacifica	Reunion	2013	ground_nester	0
PIFWS30	Ardenna_pacifica	Reunion	2013	ground_nester	0

PIFWS31	*Ardenna_pacifica*	Reunion	2013	ground_nester	0
PIFWS32	*Ardenna_pacifica*	Reunion	2013	ground_nester	0
PIFWS33	*Ardenna_pacifica*	Reunion	2013	ground_nester	0
PIFWS34	*Ardenna_pacifica*	Reunion	2013	ground_nester	0
PIFWS36	*Ardenna_pacifica*	Reunion	2013	ground_nester	0
PIFWS37	*Ardenna_pacifica*	Reunion	2013	ground_nester	0
PIFWS38	*Ardenna_pacifica*	Reunion	2013	ground_nester	0
PIFWS39	*Ardenna_pacifica*	Reunion	2013	ground_nester	0
TR-MB-011	*Sula_dactylatra*	Tromelin	2012	ground_nester	0
TR-MB-014	*Sula_dactylatra*	Tromelin	2012	ground_nester	1
TR-MB-020	*Sula_dactylatra*	Tromelin	2012	ground_nester	0
TR-MB-031	*Sula_dactylatra*	Tromelin	2012	ground_nester	0
TR-MB-032	*Sula_dactylatra*	Tromelin	2012	ground_nester	1
TR-RB-009	*Sula_sula*	Tromelin	2012	tree-nesters	0
TR-RB-018	*Sula_sula*	Tromelin	2012	tree-nesters	0
TR-RB-021	*Sula_sula*	Tromelin	2012	tree-nesters	0
TR-RB-027	*Sula_sula*	Tromelin	2012	tree-nesters	0
TR-RB-031	*Sula_sula*	Tromelin	2012	tree-nesters	0
TR-RB-033	*Sula_sula*	Tromelin	2012	tree-nesters	0
TR-RB-034	*Sula_sula*	Tromelin	2012	tree-nesters	0
TR-RB-039	*Sula_sula*	Tromelin	2012	tree-nesters	0
TR-MB-026	*Sula_dactylatra*	Tromelin	2012	ground_nester	1
TR-MB-036	*Sula_dactylatra*	Tromelin	2012	ground_nester	1
TR-MB-039	*Sula_dactylatra*	Tromelin	2012	ground_nester	0
TR-MB-040	*Sula_dactylatra*	Tromelin	2012	ground_nester	0
TR-MB-041	*Sula_dactylatra*	Tromelin	2012	ground_nester	0
TR-MB-042	*Sula_dactylatra*	Tromelin	2012	ground_nester	1
TR-MB-044	*Sula_dactylatra*	Tromelin	2012	ground_nester	1
TR-MB-045	*Sula_dactylatra*	Tromelin	2012	ground_nester	1
TR-MB-046	*Sula_dactylatra*	Tromelin	2012	ground_nester	1
TR-MB-047	*Sula_dactylatra*	Tromelin	2012	ground_nester	0
TR-MB-048	*Sula_dactylatra*	Tromelin	2012	ground_nester	1
TR-MB-050	*Sula_dactylatra*	Tromelin	2012	ground_nester	1
TR-MB-051	*Sula_dactylatra*	Tromelin	2012	ground_nester	1
TR-MB-052	*Sula_dactylatra*	Tromelin	2012	ground_nester	0
TR-RB-005	*Sula_sula*	Tromelin	2012	tree-nesters	0
TR-RB-035	*Sula_sula*	Tromelin	2012	tree-nesters	0
TR-RB-036	*Sula_sula*	Tromelin	2012	tree-nesters	0
TR-RB-040	*Sula_sula*	Tromelin	2012	tree-nesters	0

TR-RB-043	*Sula_sula*	Tromelin	2012	tree-nesters	0
TR-RB-044	*Sula_sula*	Tromelin	2012	tree-nesters	1
TR-RB-045	*Sula_sula*	Tromelin	2012	tree-nesters	0
TR-RB-046	*Sula_sula*	Tromelin	2012	tree-nesters	0
TR-RB-048	*Sula_sula*	Tromelin	2012	tree-nesters	0
TR-RB-050	*Sula_sula*	Tromelin	2012	tree-nesters	0
